# *miR-122* Deficiency in Mice Enhances Regeneration in Healthy Liver but Drives Pathological Repair and Functional Decline in Fibrotic Liver

**DOI:** 10.3390/ijms27073149

**Published:** 2026-03-30

**Authors:** Jordi Ribera, Anna Cardona-Simó, Irene Portolés, Esther Samper, Loreto Boix, Aleix B. Fabregat-Bolufer, Esther Fernández-Galán, María Rodríguez-Garcia, Mikel Azkargorta, Felix Elortza, Séverine Celton-Morizur, Chantal Desdouets, Pedro Melgar-Lesmes, Wladimiro Jiménez, Gregori Casals, Manuel Morales-Ruiz

**Affiliations:** 1 Biochemistry and Molecular Genetics Department, Hospital Clínic of Barcelona, Institut d’Investigacions Biomèdiques August Pi i Sunyer (IDIBAPS), Centro de Investigación Biomédica en Red de Enfermedades Hepáticas y Digestivas (CIBERehd), 08036 Barcelona, Spain; 2 Biomedicine Department, Faculty of Medicine and Health Sciences, University of Barcelona, 08036 Barcelona, Spain; 3 Centre de Recherche des Cordeliers, Sorbonne Université, INSERM, Université de Paris, F-75006 Paris, France; 4 Genomic Instability, Metabolism, Immunity and Liver Tumorigenesis Laboratory, Equipe Labellisée par la Ligue Nationale Contre le Cancer, F-75006 Paris, France; 5 Barcelona Clinic Liver Cancer Group, Liver Unit, Hospital Clinic, University of Barcelona, IDIBAPS, CIBERehd, 08036 Barcelona, Spain; 6 Proteomics Platform, CIC bioGUNE, Basque Research and Technology Alliance (BRTA), CIBERehd, 48160 Derio, Spain; 7 Department of Fundamental and Clinical Nursing, Faculty of Nursing, University of Barcelona, 08036 Barcelona, Spain

**Keywords:** microRNA, miR-122, liver regeneration, partial hepatectomy, fibrosis, cell proliferation, ploidy

## Abstract

MicroRNA-122 (miR-122) is the most abundant hepatic microRNA and a key regulator of hepatocyte proliferation, metabolism and differentiation. Although widely studied in hepatocellular carcinoma, its role in liver regeneration remains unexplored. This study investigated how miR-122 deficiency modulates liver regeneration under physiological conditions and during chronic liver injury. A miR-122-deficient mouse model (*miR-122^−/−^*) was generated using CRISPR/Cas9, and liver regeneration was assessed after two-thirds partial hepatectomy (PHx) in healthy and CCl_4_-induced fibrotic livers. In healthy liver, miR-122 expression was transiently downregulated within 24 h after PHx, suggesting a physiological role in cell cycle entry. After PHx in non-fibrotic livers, *miR-122^−/−^* mice showed increased basal proliferation and accelerated regeneration, associated with Cyclin D1 and RhoA overexpression, enhanced cytokinesis and a predominance of diploid hepatocytes. In contrast, miR-122 deficiency markedly exacerbated CCl_4_-induced fibrosis, leading to cirrhosis-like architecture, impaired hepatocyte function, and severe metabolic dysregulation. Despite increased proliferation after PHx, fibrotic *miR-122^−/−^* mice exhibited severely impaired regeneration and near-complete mortality. Proteomic analyses revealed metabolic failure, oxidative stress, and inflammatory activation, creating an unfavorable environment for tissue repair. In conclusion, miR-122 plays a dual role in liver regeneration. While its suppression enhances regeneration in healthy liver, loss of miR-122 under fibrotic conditions drives pathological repair, metabolic failure and lethality, highlighting its critical role in chronic liver disease.

## 1. Introduction

Liver regeneration is an evolutionarily conserved process essential for survival following chemical injury or surgical resection. This repair mechanism depends on quiescent hepatocytes re-entering the cell cycle, a transition driven by mitogenic signals (e.g., HGF, EGF) and metabolic reprogramming [1,2]. However, this regenerative potential is severely compromised in the context of chronic liver disease. In patients with advanced fibrosis or cirrhosis, functional regeneration often fails and is replaced by a maladaptive response, significantly increasing the risk of post-hepatectomy liver failure (PHLF), which remains the leading cause of mortality after major liver surgery [3,4]. Identifying the molecular factors that distinguish functional regeneration from pathological failure is therefore a critical clinical need.

MicroRNAs (miRNAs) are key post-transcriptional regulators of liver health and disease [5,6,7,8]. Among them, miR-122 is the most abundant, making up nearly 70% of the total miRNA pool in the adult liver [9]. Beyond its well-established roles in regulating lipid metabolism, iron homeostasis, and hepatitis C virus replication [5,6,10], miR-122 acts as a tumor suppressor [10,11,12,13]; its loss is a hallmark of hepatocellular carcinoma and is associated with poor prognosis [6,14]. Furthermore, germline deletion of *miR-122* in mice results in spontaneous steatohepatitis and progressive fibrosis, underscoring its role in maintaining hepatocyte identity and suppressing inflammation [13,15].

Despite these advances, the functional kinetics of miR-122 during the acute phase of liver regeneration remain unclear. While it is known that microRNAs are critical regulators of hepatocyte cell cycle progression during liver regeneration [16], the precise contribution of miR-122 to the regulation of proliferation is not fully understood. Moreover, although miR-122 is consistently downregulated in human cirrhosis [17], it is unknown whether this loss is merely a marker of the disease or a driver of the catastrophic regenerative failure observed in fibrotic livers.

In this study, we use a germline *miR-122* knockout (*miR-122^−/−^*) mouse model to investigate the role of this microRNA in liver repair under both physiological and pathological conditions. Here, we hypothesize that miR-122 helps balance proliferative signals with metabolic capacity and we demonstrate that while miR-122 deficiency accelerates regeneration in the healthy liver by overexpressing cell cycle regulators like Cyclin D1 and RhoA, its absence in a fibrotic background triggers an uncontrolled increase in the fibrosis, a fatal bioenergetic collapse, and loss of hepatic function. These findings suggest a context-dependent duality for miR-122 and identify its restoration as a potential therapeutic strategy to prevent liver failure in surgical patients with underlying liver disease.

## 2. Results

### 2.1. Germline miR-122 Knockout Mouse Presents Accelerated Proliferation and Regeneration

**Figure 1:**
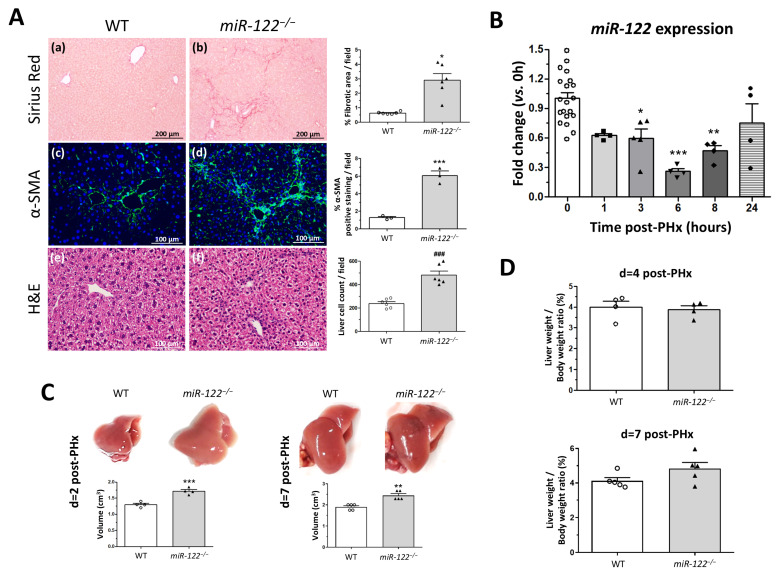
*miR-122* downregulation triggers liver regeneration. (**A**) Representative Sirius Red (panels (**a**,**b**)), α-SMA staining (panels (**c**,**d**)) and hematoxylin and eosin (H&E) (panels (**e**,**f**)) stained liver sections from wild-type (WT) mice and *miR-122* knockout (*miR-122^−/−^*) mice. Original magnification: ×100 for Sirius Red staining and ×200 for α-SMA and H&E staining. Computer-assisted quantifications for the percentage of fibrotic area and liver cell count are shown in the right graphs. Bars represent the mean ± SEM; * *p* = 0.018, *** *p* = 0.0008 and ^###^ *p* = 0.0001 vs. wild-type mice (*n* = 6 for Sirius Red and H&E (3 males and 3 females), and *n* = 3 for α-SMA staining (2 males and 1 female)). (**B**) Liver tissue from WT mice after partial hepatectomy (PHx) at different time points was lysed in trizol and total mRNA was extracted. *miR-122* expression was analyzed by RT-qPCR using Taqman microRNA assay Hsa-miR-122, as described in Methods. Graph shows the expression levels for *miR-122* as fold change relative to *Rnu6b* levels. Bars represent the mean ± SEM; * *p* = 0.015, ** *p* = 0.002 and *** *p* = 0.0001 vs. time 0 h (*n* = 4 biologically independent samples for each time point, 2 males and 2 females). (**C**) Hepatic volume obtained in WT and *miR-122^−/−^* mice 2 and 7 days after PHx. Bars represent the mean ± SEM; ** *p* = 0.0013 and *** *p* = 0.0007 vs. WT mice (*n* = 4 for day 2 (2 males and 2 females) and *n* = 5 for day 7 (3 males and 2 females)). (**D**) Hepatic regenerative index (liver weight/body weight ratio) obtained in WT and *miR-122^−/−^* mice 4 and 7 days after PHx. Bars represent the mean ± SEM (*n* = 5, 2 males and 3 females).

*miR-122^−/−^* mice were born healthy and fertile and displayed no apparent morphological differences compared with wild-type mice. To evaluate the physiological status of the liver of *miR-122^−/−^* mice, Sirius Red staining was performed to analyze the fibrotic state of young *miR-122^−/−^* mice. Our three-month-old *miR-122^−/−^* mice displayed spontaneous mild liver fibrosis in the basal state, with approximately 3% of the liver area occupied by fibrotic tissue (Figure 1A, panels a and b). α-SMA expression was also quantified as a marker of activated hepatic stellate cells, revealing higher levels in *miR-122^−/−^* livers compared with WT controls (6.07 ± 0.51 vs. 1.28 ± 0.12% α-SMA-positive staining per field, respectively; *p* = 0.0008) (Figure 1A, panels c and d). Hematoxylin and eosin (H&E) histological staining was also analyzed, we observed a significant two-fold increase in the number of cells in *miR-122^−/−^* livers compared with wild-type (WT) livers (483.30 ± 60.09 vs. 241.70 ± 22.05 liver cell count per field, respectively; *p* = 0.0001) (Figure 1A, panels e and f), while liver size remained the same between the two phenotypes.

To study the role of miR-122 during liver regeneration, we performed two-thirds partial hepatectomy (PHx) on WT and *miR-122^−/−^* mice. First, a time-course analysis of miR-122 expression in WT mice after PHx showed a rapid and significant decrease in miR-122 levels within one hour post-surgery, reaching its lowest point at six hours, before normalizing at 24 h (Figure 1B). This transient downregulation suggests that the acute decrease in miR-122 is a prerequisite signal required to trigger the transcriptional cascade for hepatocyte entry into the cell cycle.

Consistent with this requirement, *miR-122^−/−^* mice showed accelerated regeneration. Two days after PHx, *miR-122^−/−^* mice had visibly larger and more voluminous livers compared to WT mice (1.72 ± 0.07 vs. 1.30 ± 0.06 cm^3^, respectively; *p* = 0.0007), indicating an advanced regenerative state. This increase in total liver volume continued to be observed on day 7 (2.44 ± 0.09 vs. 1.90 ± 0.06 cm^3^, respectively; *p* = 0.0013) (Figure 1C). Regarding the liver-to-body weight ratio, at day 4 after PHx, it was similar between *miR-122^−/−^* and WT mice (3.88 ± 0.19 vs. 4.00 ± 0.29, respectively; *p* = n.s.), and by day 7 both groups reached the endpoint of regeneration, showing also equivalent ratios between groups (Higgins index: 4.83 ± 0.36 vs. 4.12 ± 0.19, respectively; *p* = n.s.) (Figure 1D). These results suggest that the absence of miR-122 accelerates the rate of regeneration without causing liver overgrowth.

**Figure 2:**
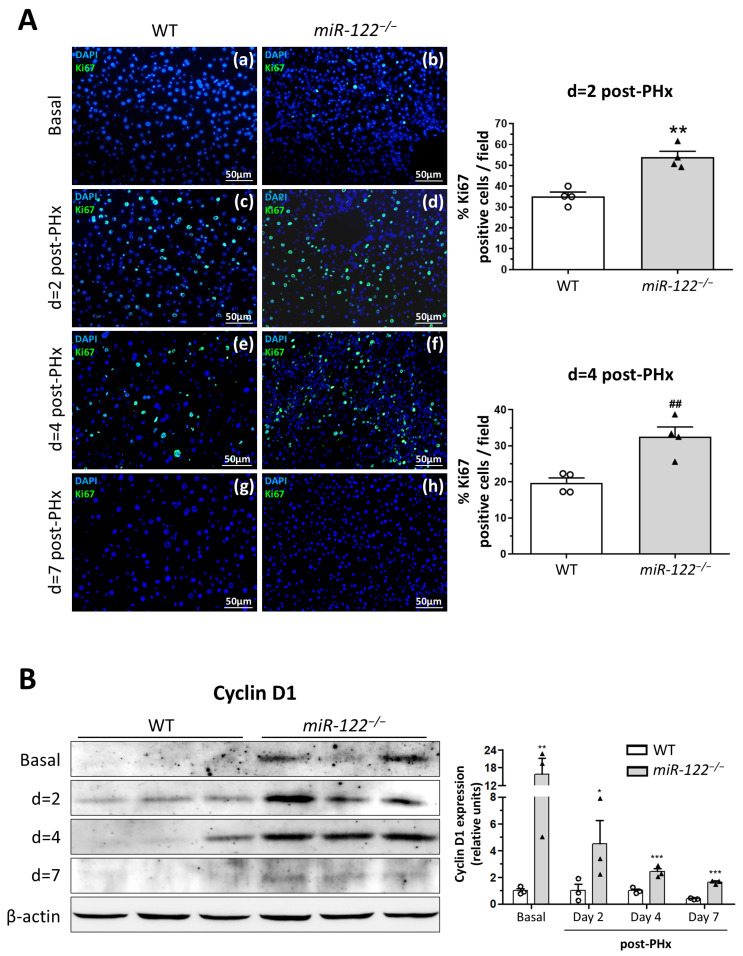
Absence of *miR-122* enhances cell proliferation and liver regeneration. (**A**) Representative merged images of immunofluorescence staining of Ki67-positive cells (green) and DAPI (blue) in wild-type (WT) and *miR-122^−/−^* livers at basal (**a**,**b**), 2 days (**c**,**d**), 4 days (**e**,**f**) and 7 days (**g**,**h**) following partial hepatectomy (PHx). Original magnification ×200. Graphs show the computer-assisted quantification of Ki67-positive cells/total nuclei at different times following PHx. Bars represent mean ± SEM, ** *p* = 0.0016 and ^##^ *p* = 0.005 vs. wild-type (*n* = 4). (**B**) The expression of Cyclin D1 protein was evaluated by Western blot using liver tissue lysates from WT and *miR-122^−/−^* mice at different points after PHx. β-actin was used as a loading control. The densitometric analysis of the protein expression is shown in the right graph. Bars represent the mean ± SEM; * *p* = 0.040, ** *p* = 0.0016, *** *p* = 0.0001 vs. wild-type mice (*n* = 3).

Ki67 immunostaining confirmed enhanced cell proliferation, with *miR-122^−/−^* mice showing mild proliferative activity at baseline, while WT livers displayed almost no proliferating cells (Figure 2(Aa,Ab)). Two days after PHx, regenerating *miR-122^−/−^* livers exhibited markedly higher proliferation compared to WT mice (53.96 ± 5.63 vs. 35.19 ± 2.29% Ki67 positive cells per field, respectively; *p* = 0.0016) (Figure 2(Ac,Ad)). This state of increased proliferation was maintained on day 4 (32.57 ± 3.81 vs. 19.70 ± 1.42% Ki67 positive cells per field, respectively; *p* = 0.005) (Figure 2(Ae,Af)) and on day 7 after PHx, neither the WT nor *miR-122^−/−^* mice showed any Ki67 positive cell (Figure 2(Ag,Ah)), consistent with the similar Higgins index values observed at this point.

### 2.2. miR-122 Loss Upregulates Cyclin D1 and RhoA, Driving Diploidy

**Figure 3:**
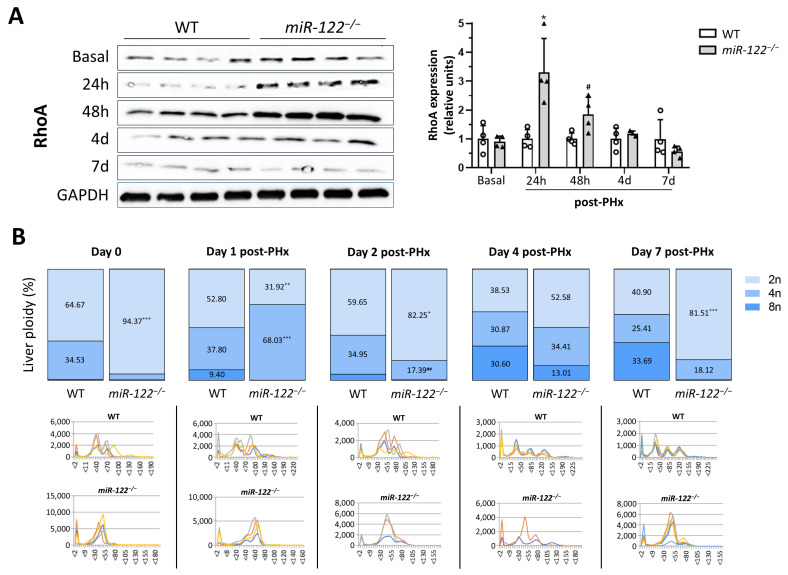
*miR-122* suppression increases RhoA activity and cytokinesis. (**A**) The expression of RhoA protein was evaluated by Western blot using liver tissue lysates from wild-type (WT) and *miR-122* knockout (*miR-122^−/−^*) mice at different points after partial hepatectomy (PHx). GAPDH was used as a loading control. The densitometric analysis of the protein expression is shown in the right graph. Bars represent the mean ± SEM; * *p* = 0.012 and ^#^ *p* = 0.035 vs. WT mice (*n* = 4). (**B**) Evaluation of the hepatic nuclear ploidy profiles from WT and *miR-122^−/−^* mice at different time points after PHx. Bottom graphs represent the number of cells of the different populations located between areas of 30–60 pixel^2^, 65–90 pixel^2^ and 95–500 pixel^2^ to separate the 2n, 4n and ≥8n nuclei, respectively; * *p* = 0.034, ** *p* = 0.0016, ^##^ *p* = 0.005, *** *p* = 0.0001 vs. WT mice (*n* = 4 for each time point).

Western blot analysis showed that *miR-122^−/−^* mice exhibited basal and sustained significant overexpression of Cyclin D1 compared to WT mice at days 2, 4 and 7 after PHx (Figure 2B). In contrast, PCNA and Cyclin G1, a described direct target of miR-122 [18], showed no significant or consistent differences in our model (Supplementary Figure S1A,B). The established target of miR-122, RhoA, a well-known key regulator of the cytokinetic process and cell ploidy [19,20], was also found to be significantly overexpressed in *miR-122^−/−^* livers at 24 and 48 h after PHx (Figure 3A).

Analysis of hepatic ploidy revealed that in adult WT livers, the cell population included diploid (2n) and tetraploid (4n, binucleate) cells. In contrast, *miR-122^−/−^* livers were predominantly diploid (64.67 ± 2.25 vs. 94.37 ± 0.78% diploid cells per field, respectively; *p* = 0.0001). Following partial hepatectomy, at 24 h, both genotypes showed a decrease in diploid cells and an increase in tetraploid cells, but these changes were more pronounced in the *miR-122^−/−^* livers (52.80 ± 1.69 vs. 31.92 ± 1.70% diploid cells per field, respectively; *p* = 0.0016, and 37.80 ± 0.38 vs. 68.03 ± 1.70% tetraploid cells per field, respectively; *p* = 0.0001). By day 2, WT livers maintained a substantial tetraploid population, while KO livers recovered most of their diploid state (59.65 ± 6.19 vs. 82.25 ± 3.11% diploid cells per field, respectively; *p* = 0.034, and 34.95 ± 2.26 vs. 17.39 ± 3.04% tetraploid cells per field, respectively; *p* = 0.005). On day 4, WT cells were distributed approximately equally among the 2n, 4n, and 8n populations, while KO livers remained primarily diploid (38.53 ± 1.44 vs. 52.58 ± 16.20% diploid cells per field, respectively; *p* = n.s.). On day 7, WT livers showed again a predominance of diploid cells with some 4n and 8n cells, while KO livers retained a mostly diploid population (40.90 ± 1.71 vs. 81.51 ± 4.99% diploid cells per field, respectively; *p* = 0.0001) (Figure 3B), suggesting enhanced cytokinesis efficiency and accelerated cell division and regeneration, likely due to RhoA overexpression in the absence of miR-122.

### 2.3. miR-122 Expression Is Reduced in Liver Fibrosis, and Its Absence Exacerbates Chronic CCl_4_-Induced Injury

**Figure 4:**
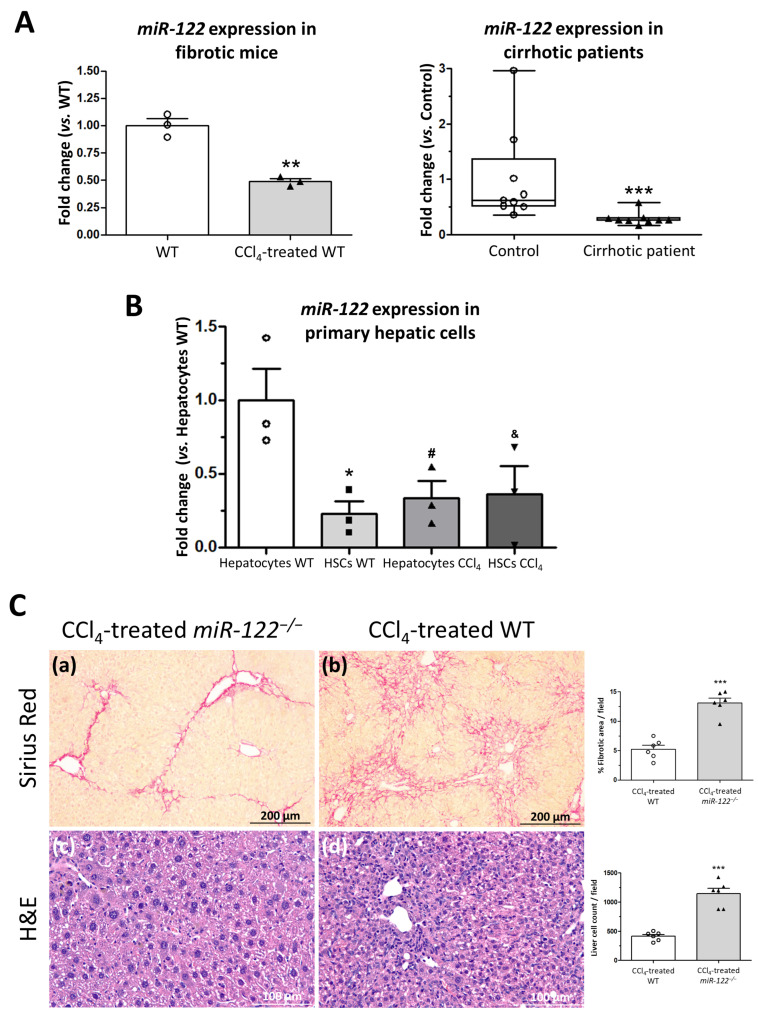
Loss of *miR-122* exacerbates the liver fibrotic process. (**A**) Liver tissue from wild-type (WT) mice and CCl_4_-treated mice (*n* = 3), and from control and cirrhotic patients (*n* = 9) was lysed in trizol and total mRNA was extracted. *miR-122* expression was analyzed by RT-qPCR using Taqman microRNA assay Hsa-miR-122, as described in Methods. Graphs show the expression levels for *miR-122* as fold change relative to *RNU6B* levels. In the left graph, bars represent the mean ± SEM (unpaired *t*-test), and in the right graph, bars represent the median and IQR with whiskers indicating the minimum and maximum values (Mann–Whitney U test); ** *p* = 0.0014 and *** *p* = 0.0003 vs. WT mice/human non-cirrhotic samples. (**B**) Total mRNA from isolated hepatocytes and hepatic stellate cells (HSCs) from wild-type (WT) mice and CCl_4_-treated mice (*n* = 3) was extracted using trizol. *miR-122* expression was analyzed by RT-qPCR using Taqman microRNA assay Hsa-miR-122, as described in Methods. Graph shows the expression levels for *miR-122* as fold change relative to *RNU6B* levels. Bars represent the mean ± SEM; * *p* = 0.018, ^#^ *p* = 0.037 and ^&^ *p* = 0.043 vs. hepatocytes WT. (**C**) Representative Sirius Red (**a**,**c**) and hematoxylin and eosin (H&E) (**b**,**d**) stained liver sections from CCl_4_-treated WT mice and CCl_4_-treated *miR-122^−/−^* mice. Original magnification: ×100 for Sirius Red staining and ×200 for H&E staining. Computer-assisted quantifications for the percentage of fibrotic area and liver cell count are shown in the bottom graphs. Bars represent the mean ± SEM; *** *p* = 0.0001 vs. CCl_4_-treated WT mice (*n* = 6, 3 males and 3 females).

We next investigated the role of miR-122 in chronic liver injury. Liver fibrosis is known to be associated with reduced miR-122 levels [17], and we confirmed this significant decrease in both CCl_4_-treated WT mice (two-fold decreased) and in human cirrhotic patient samples (three-fold decreased) compared to their respective non-cirrhotic samples (Figure 4A). To determine which cell types are responsible for the reduction in miR-122 in fibrosis, we analyzed its expression in primary hepatocytes and hepatic stellate cells (HSCs) isolated from both untreated and CCl_4_-treated mice. In healthy livers, hepatocytes were the main source of miR-122, while HSCs expressed very low levels (five-fold decreased). In CCl_4_-induced fibrosis, *miR-122* expression decreased by nearly 70% in hepatocytes, reaching levels comparable to those observed in HSCs, which remained largely unchanged, indicating that the loss of miR-122 in the fibrotic liver is primarily due to a hepatocyte downregulation (Figure 4B).

When *miR-122^−/−^* mice were subjected to the CCl_4_ treatment, they developed severe fibrosis compared with WT mice. Sirius Red staining and histological analysis revealed an exacerbated extracellular matrix accumulation, reaching up to 13% of the total liver area, with dense, fused fibrotic septa resembling cirrhosis (13.13 ± 0.80 vs. 5.23 ± 0.68% fibrotic area per field, respectively; *p* = 0.0001) (Figure 4(Ca,Cb)). Histological examination also confirmed a dramatic increase in the cell number of *miR-122^−/−^* fibrotic livers compared to WT fibrotic controls (1147.00 ± 90.14 vs. 414.60 ± 38.08 liver cell count per field, respectively; *p* = 0.0001) (Figure 4(Cc,Cd)). Unlike in the healthy context, the size of the livers was significantly larger in the KO fibrotic mice compared to WT fibrotic mice (1.27 ± 0.08 vs. 0.63 ± 0.04 g of liver left lobe weight, respectively; *p* = 0.0001) (Supplementary Figure S2). This demonstrates that the absence of miR-122 significantly sensitizes the liver to chemical injury, accelerating and exacerbating the fibrotic process.

### 2.4. Loss of miR-122 Leads to Pathological Regeneration and Increased Mortality Following PHx in Fibrotic Livers

**Figure 5:**
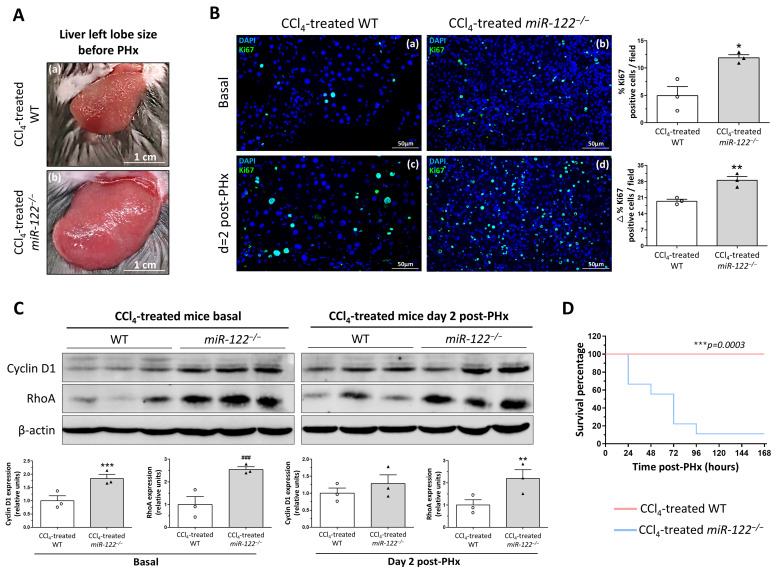
Absence of *miR-122* enhances cell proliferation but increases mortality during liver regeneration in a fibrotic context. (**A**) Representative images of the liver left lobe from a CCl_4_-treated wild-type (WT) mouse (**a**) and a CCl_4_-treated *miR-122* knockout (*miR-122^−/−^*) mouse (**b**) before partial hepatectomy. (**B**) Representative merged images of immunofluorescence staining of Ki67-positive cells (green) and DAPI (blue) in CCl_4_-treated WT and CCl_4_-treated *miR-122^−/−^* livers at basal (**a**,**b**) and 2 days (**c**,**d**) following partial hepatectomy (PHx). The quantification of Ki67-positive cells at day 2 was corrected by subtracting the basal time percentage (△%Ki67). Original magnification ×200. Graphs on the right show the computer-assisted quantification of Ki67-positive cells/total nuclei. Bars represent mean ± SEM, * *p* = 0.0017 and ** *p* = 0.009 vs. CCl_4_-treated WT mice (*n* = 3, 2 males and 1 female). (**C**) The expression of Cyclin D1 and RhoA proteins was evaluated by Western blot using liver tissue lysates from CCl_4_-treated WT and CCl_4_-treated *miR-122^−/−^* mice at basal time and 2 days after PHx. β-actin was used as a loading control. The densitometric analysis of the protein expression is shown in the bottom graphs. Bars represent the mean ± SEM; ** *p* = 0.0018, *** *p* = 0.0003 and ^###^ *p* = 0.0001 vs. CCl_4_-treated WT mice (*n* = 3, 1 male and 2 females). (**D**) Survival graph showing CCl_4_-treated WT and CCl_4_-treated *miR-122^−/−^* mice mortality from 24 h to 7 days after PHx. CCl_4_-treated *miR-122^−/−^* mice showed a clear significant lower survival; *** *p* = 0.0003 vs. CCl_4_-treated WT mice (*n* = 9, 5 males and 4 females).

Fibrotic *miR-122^−/−^* hepatic lobes were significantly larger, more than twice the size of WT fibrotic hepatic lobes, and exhibited a rigid texture (Figure 5A), reflecting an uncontrolled fibrogenic response even at baseline. In this context, we examined the proliferative response after the PHx. Basal proliferation, measured by Ki67, was already significantly higher in *miR-122^−/−^* fibrotic livers compared with WT fibrotic controls (11.89 ± 0.57 vs. 4.96 ± 1.68% Ki67 positive cells per field, respectively; *p* = 0.017) (Figure 5(Ba,Bb)). Given the marked proliferation observed at baseline, these values were subtracted from those measured on day 2. This normalization strategy effectively isolates the PHx-specific mitogenic response from the background compensatory proliferation inherent to the fibrotic environment. In this context, two days after the surgery, proliferation remained elevated in *miR-122^−/−^* livers relative to WT livers (28.53 ± 1.71 vs. 19.42 ± 0.83% Ki67 positive cells per field, respectively; *p* = 0.009) (Figure 5(Bc,Bd)). In correlation with the healthy context, Cyclin D1 levels were significantly increased in *miR-122^−/−^* fibrotic livers at baseline, promoting the entry of hepatic cells into the cell cycle. RhoA expression also remained elevated, in this case both at baseline and two days post-PHx (Figure 5C), suggesting persistent dysregulation of the cytokinesis process and an accelerated cell division.

Despite this enhanced proliferative activity, *miR-122^−/−^* mice undergoing PHx in the fibrotic setting exhibited nearly 100% mortality shortly after surgery (Figure 5D). This critical and unexpected outcome demonstrates that, despite increased proliferation, regeneration was severely impaired, ultimately leading to acute liver failure and lethality.

### 2.5. Impaired Hepatocyte Function and Altered Cellular Composition During Regeneration in Fibrotic miR-122^−/−^ Livers

**Figure 6:**
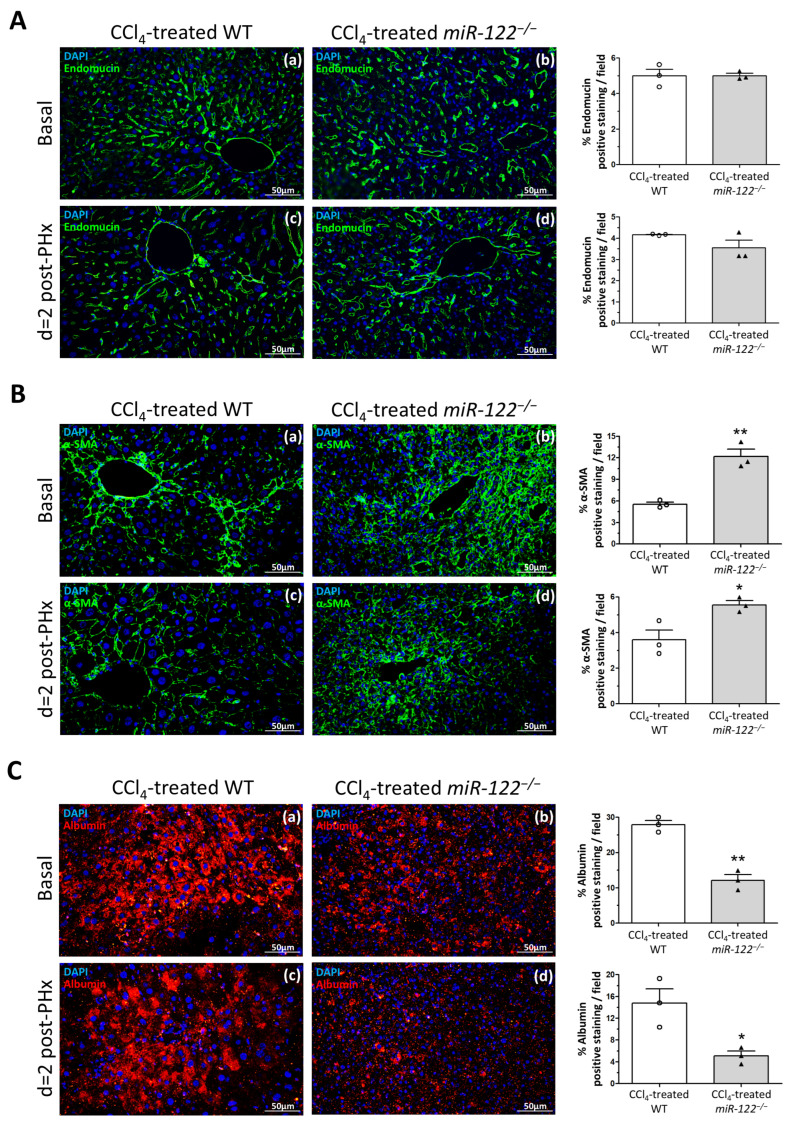
The absence of *miR-122* impairs hepatic function during liver regeneration. (**A**) Endomucin immunostaining of liver vessels and hepatic sinusoids in CCl_4_-treated wild-type (WT) and CCl_4_-treated *miR-122* knockout (*miR-122^−/−^*) mice at basal (panels (**a**,**b**)) and 2 days (panels (**c**,**d**)) following partial hepatectomy (PHx). Original magnification: 200×. Quantification of the percentage of endomucin positive staining is shown in the right graphs (*n* = 3). (**B**) α-smooth muscle actin (α-SMA) immunostaining of activated hepatic stellate cells in CCl_4_-treated WT and CCl_4_-treated *miR-122^−/−^* mice at basal (panels (**a**,**b**)) and 2 days (panels (**c**,**d**)) following PHx. Original magnification: 200×. Quantification of the percentage of α-SMA-positive staining is shown in the right graphs. Bars represent the mean ± SEM; * *p* = 0.031 and ** *p* = 0.003 vs. CCl_4_-treated WT mice (*n* = 3). (**C**) Albumin immunostaining of functional hepatocytes in CCl_4_-treated WT and CCl_4_-treated *miR-122^−/−^* mice at basal (**a**,**b**) and 2 days (panels (**c**,**d**)) following PHx. Original magnification: 200×. Quantification of the percentage of albumin positive staining is shown in the right graphs. Bars represent the mean ± SEM; * *p* = 0.023 and ** *p* = 0.0014 vs. CCl_4_-treated WT mice (*n* = 3).

Given the increased number of Ki67-positive nuclei on day 2 post-surgery, we next assessed whether non-parenchymal cell populations contributed to this proliferative signal. Endomucin immunostaining was used to evaluate endothelial cells. No differences in vascular density or endomucin-positive structures were detected between WT and *miR-122^−/−^* fibrotic livers, either at baseline or at day 2 after PHx (Figure 6A).

To assess hepatic stellate cell content, alpha-smooth muscle actin (α-SMA) staining was performed. *miR-122^−/−^* fibrotic livers displayed a higher abundance of α-SMA-positive cells at baseline compared with WT, consistent with their increased fibrotic area (12.18 ± 1.03 vs. 5.56 ± 0.29% α-SMA-positive staining per field, respectively; *p* = 0.003) (Figure 6(Ba,Bb)). These differences persisted at day 2 post-PHx, with *miR-122^−/−^* livers maintaining elevated α-SMA staining relative to WT (5.54 ± 0.24 vs. 3.59 ± 0.55% α-SMA-positive staining per field, respectively; *p* = 0.031) (Figure 6(Bc,Bd)). The persistence of a high α-SMA-positive cell population indicates that a substantial fraction of the proliferating (Ki67-positive) cells in *miR-122^−/−^* livers correspond to hepatic stellate cells, resulting in a proliferative profile distinct from that of WT livers.

Hepatocyte function was finally examined by albumin immunostaining. WT fibrotic livers showed strong albumin expression at baseline and maintained detectable levels on day 2 after PHx (Figure 6(Ca,Cc)). In contrast, *miR-122^−/−^* fibrotic livers exhibited markedly reduced albumin staining at baseline, and albumin was almost absent at day 2 (27.89 ± 1.33 vs. 12.15 ± 1.60% albumin positive staining per field at day 0, respectively; *p* = 0.0014, and 14.82 ± 2.58 vs. 5.11 ± 0.87% albumin positive staining per field at day 2, respectively; *p* = 0.023) (Figure 6(Cb,Cd)), indicating a pronounced loss of hepatocyte functional capacity during regeneration.

### 2.6. Pharmacological Silencing of miR-122 in CCl_4_-Treated WT Mice Validates Its Direct Contribution to Impaired Liver Function and Survival After PHx

Based on the aforementioned findings, we aimed to determine whether the observed hepatic dysfunction and increased mortality in *miR-122^−/−^* mice were driven primarily by the severity of the underlying fibrosis, given that CCl_4_-treated *miR-122^−/−^* mice developed approximately three-fold more fibrosis than their WT counterparts, or if these alterations were directly influenced by *miR-122* deficiency itself. To decouple these factors, we partially silenced miR-122 in WT mice during the establishment of CCl_4_-induced injury prior to partial hepatectomy using a specific anti-miR-122 (mmu-miR-122-5p *mir*Vana™).

In this experimental setting, unlike the *miR-122^−/−^* mouse model, both groups started with comparable livers in terms of size and baseline fibrosis (Supplementary Figure S3A). Molecular analysis confirmed a significant ~30% reduction in *miR-122* expression levels compared to the scramble control group (0.72 ± 0.01 vs. 1.00 ± 0.08 expression of *miR-122*, respectively; *p* = 0.029) (Supplementary Figure S3B). Regarding the regenerative capacity at day 7 post-PHx, no significant differences were detected in the liver-to-body weight ratio between groups. Similarly, Ki67 immunostaining revealed no significant differences in cell proliferation at baseline or at day 2 post-surgery (Supplementary Figure S3C,D).

These observations were complemented by analysis of α-SMA expression as a marker of activated hepatic stellate cells. While baseline α-SMA levels were equivalent between groups (confirming similar starting fibrosis), WT control mice showed a significant reduction in fibrosis by day 2 post-PHx, consistent with effective tissue remodeling and functional regeneration. In contrast, miR-122-silenced livers maintained significantly higher fibrotic levels (11.68 ± 1.51 vs. 6.76 ± 0.29% α-SMA-positive area, respectively; *p* = 0.033; Supplementary Figure S3E), indicating impaired fibrosis resolution. Furthermore, miR-122-silenced mice exhibited a trend toward increased mortality, although this did not reach statistical significance (*p* = 0.308; Supplementary Figure S3F).

Overall, these results partially recapitulate the *miR-122^−/−^* mouse phenotype and demonstrate that miR-122 deficiency directly contributes to impaired liver repair and reduced survival, independent of the initial severity of fibrosis.

### 2.7. Proteomic Profiling Reveals Extensive Molecular Dysregulation in miR-122^−/−^ Livers Under Non-Fibrotic and Fibrotic Conditions

To further investigate the mechanisms underlying the loss of hepatic function in *miR-122^−/−^* mice, we performed a proteomic analysis comparing knockout and wild-type livers at day 2 post-PHx under both non-fibrotic and fibrotic conditions, followed by pathway and network interrogation using the Ingenuity Pathway Analysis (IPA; Ingenuity) database. Differentially abundant proteins were analyzed separately for each context to identify the most significantly affected biological networks and disease-associated functions.

**Figure 7:**
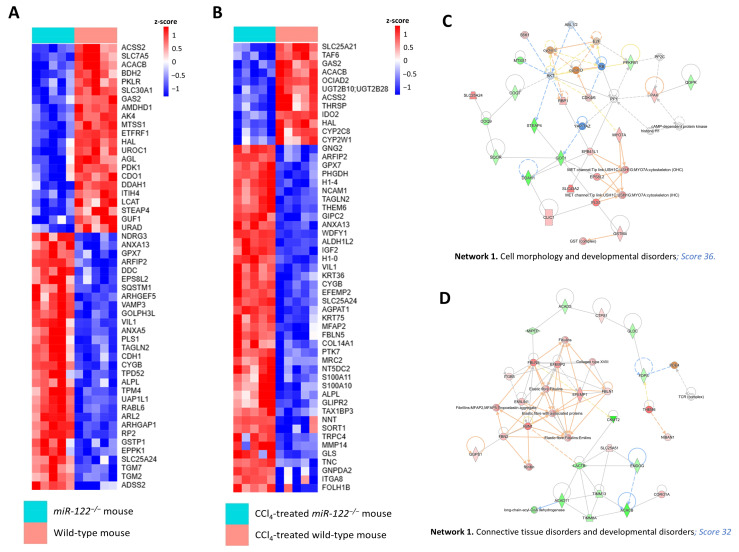
Bioinformatic analysis of the proteome from *miR-122* knockout mouse in both healthy and fibrotic contexts. (**A**,**B**) Hierarchical clustering analysis of differentially regulated proteins found in the wild-type (WT) and the *miR-122* knockout (*miR-122^−/−^*) livers (**A**) or in the CCl_4_-treated WT and the CCl_4_-treated *miR-122^−/−^* livers (**B**), 2 days after partial hepatectomy (PHx). Red pixels correspond to an increased abundance of protein and blue pixels correspond to a decreased abundance of protein, in the indicated sample. The intensity of each color denotes the standardized ratio between each value and the average expression of each protein across all samples. Z-score values are represented in the red–blue code bar shown on the right (*n* = 5). (**C**,**D**). Networks of pathways obtained from the proteomic analysis of WT and *miR-122^−/−^* livers (**C**) or CCl_4_-treated WT and the CCl_4_-treated *miR-122^−/−^* livers (**D**), 2 days after PHx, were algorithmically generated based on their connectivity. The proteins are represented as nodes, and the biological relationship between two nodes is represented as an edge (line). A colored node indicates a protein that was detected by the proteomic screening (red: overexpressed, green: reduced expression, orange: predicted activation and blue: predicted inhibition in *miR-122^−/−^* liver). Intensity of the color indicates the magnitude of expression change or the confidence of the prediction. Nodes are displayed using various shapes that represent the functional class of the proteins. Edges with dashed lines show indirect interaction.

Differential protein abundance analysis revealed widespread and statistically significant proteomic alterations between genotypes under both non-fibrotic and fibrotic conditions (*q* < 0.05, |log2FC| ≥ 0.5) (Supplementary Figure S4A,B). Unsupervised hierarchical clustering of the most stringently regulated proteins, visualized as heatmaps, showed a clear separation between knockout and wild-type samples in both contexts, indicating distinct and reproducible proteomic signatures associated with miR-122 deficiency (Figure 7A,B). Given these distinct proteomic signatures, subsequent analyses were performed separately for the non-fibrotic and fibrotic contexts.

In the non-fibrotic context, *miR-122^−/−^* livers displayed broad and coordinated molecular dysregulation. Proteomic changes (Supplementary Table S1) included early bioenergetic alterations, such as reduced mitochondrial proteins involved in oxidative phosphorylation and mitochondrial translation (ETFRF1, GUF1) and downregulation of glycolytic enzymes (PKLR, PDK1). Lipid metabolism was also affected, with decreased fatty acid synthesis and impaired regulation of β-oxidation (ACSS2, ACACB) alongside increased membrane-remodeling proteins (ANXA5, ANXA13), indicating compromised membrane integrity. Additionally, miR-122 deficiency promoted activation of proliferative pathways (RABL6, TPD52), cytoskeletal remodeling (TAGLN2, TPM4, VIL1), and heightened inflammatory signaling (SQSTM1, GPX7), reflecting a metabolically unstable, pro-inflammatory, and hyperproliferative regenerative state. IPA network analysis identified several enriched biological networks, among which “Cell morphology and developmental disorder” (score 36) (Figure 7C) and “Cell death and survival and tissue morphology” (score 20) (Supplementary Figure S5A) were notably affected, highlighting alterations in cellular structure, tissue organization, and viability.

In the fibrotic context, *miR-122^−/−^* livers exhibited a distinct and more severe proteomic signature (Supplementary Table S2). We observed a pronounced bioenergetic collapse, reflected by upregulation of key mitochondrial metabolism proteins (NNT, SLC25A24, ALDH1L2), indicating a complex remodeling of mitochondrial function and energetic imbalance. This was accompanied by decreased protein synthesis capacity, reflected by downregulation of transcriptional and chromatin-associated regulators (TAF6, ACSS2). There was robust upregulation of extracellular matrix proteins (COL14A1, EFEMP2, FBLN5, MFAP2) indicating persistent and excessive matrix deposition. Furthermore, fibrotic livers displayed augmented proliferative signaling (IGF2, PTK7), cytoskeletal remodeling (VIL1), and increased oxidative and redox imbalance (GPX7, CYGB). Epigenetic dysregulation was also observed, including changes in multiple histone variants (H1-4, H1-0), collectively reflecting a severely impaired regenerative response. IPA network analysis also identified several highly enriched networks, among which “Connective tissue disorders and developmental disorders” (score 32) (Figure 7D) and “Connective tissue development and function, embryonic development and organ morphology” (score 18) (Supplementary Figure S5B) were particularly relevant, underscoring extracellular matrix remodeling, tissue fibrosis, and dysregulated developmental pathways.

IPA diseases and function analysis revealed a largely overlapping functional signature between the two contexts, with proteomic alterations affecting cancer, metabolic disease, cell death and survival, and cellular function and maintenance, reflecting consistent impacts on fundamental cellular processes. Notably, the fibrotic context showed additional features indicative of disease progression, including connective tissue disorders and enhanced inflammatory processes, as evidenced by enrichment of inflammatory disease (Supplementary Figure S6B), whereas the non-fibrotic context was characterized by a relatively broader representation of homeostatic and developmental functions (Supplementary Figure S6A).

## 3. Discussion

This study investigates the role of miR-122 in liver regeneration under physiological and fibrotic conditions using a germline *miR-122* knockout mouse model that we generated. Notably, this model recapitulates several key phenotypic features previously described by Hsu et al. [13] and Tsai et al. [15], including metabolic dysregulation, enhanced inflammatory signaling, and increased vulnerability to liver injury. These established characteristics provide strong validation of our model and offer a biologically coherent framework for interpreting the broad regenerative and metabolic phenotypes observed in our study.

This study identifies miR-122 as a central regulator of regenerative capacity, ploidy, and metabolic stability, revealing distinct roles for this microRNA in healthy and fibrotic contexts. Using a germline *miR-122* knockout model, we show that the absence of miR-122 accelerates liver regeneration under physiological conditions but leads to severely impaired outcomes and high mortality when regeneration occurs on a fibrotic background. These findings demonstrate that miR-122 acts as a molecular brake on proliferation in the healthy liver and becomes essential for preserving tissue integrity and functional regeneration during chronic injury.

In healthy liver, miR-122 deficiency resulted in a significant increase in basal proliferation and an accelerated regenerative response following partial hepatectomy. This hyperproliferative state was associated with elevated expression of Cyclin D1 and RhoA, two key regulators of G1–S progression and cytokinesis [21]. The transient decrease in miR-122 observed in WT mice immediately after partial hepatectomy suggests that physiological downregulation of this microRNA is an essential early event that enables hepatocytes and other liver cell populations to re-enter the cell cycle. As miR-122 acts as a brake that integrates growth-inhibitory signals to prevent cell cycle progression [22,23], its deletion removes this checkpoint entirely, resulting in an accelerated regeneration without leading to pathological overgrowth. Although Cyclin G1 has been described as a direct target of miR-122 [18], it did not show consistent differences in our model, supporting the idea that dysregulated Cyclin D1 and RhoA are the predominant drivers of the hyperproliferative phenotype. Despite the increase in Cyclin D1 expression, we cannot discard that this effect is an indirect consequence of the absence of miR-122.

miR-122 loss also deeply altered ploidy dynamics, shifting the balance from a mixture of diploid, tetraploid, and octoploid hepatocytes found in adult wild-type livers toward a marked enrichment of diploid cells in *miR-122^−/−^* livers, both at baseline and throughout regeneration. This observation is concordant with prior reports that miR-122 is required for the normal polyploidization program in mouse liver [19] and suggests that elevated RhoA and improved cytokinesis efficiency shift divisions toward productive cytokinesis and diploidy rather than polyploidization. Because hepatocyte ploidy is linked to metabolic capacity, stress resilience and genomic stability, the failure to acquire normal polyploid states may contribute to altered functional outcomes in miR-122-deficient livers [19]. While this diploid state facilitates rapid tissue expansion in healthy mice, the loss of polyploidy may reduce the liver’s ability to buffer against oxidative stress, as previously described in the non-alcoholic fatty liver disease context [24].

miR-122 deficiency dramatically worsened fibrosis after CCl_4_ exposure. The extent and severity of fibrotic remodeling in this model significantly exceed those typically achieved in classical murine fibrosis models [25,26,27,28], which rarely progress beyond advanced fibrosis. In contrast, *miR-122^−/−^* mice exhibited a remarkable cirrhosis-like phenotype characterized by extensive extracellular matrix deposition, abundant α-SMA-positive hepatic stellate cell activation, and a pronounced expansion in total cell number. Together, these features resulted in a liver architecture closely resembling human cirrhosis, underscoring the unique value of this model for studying end-stage fibrotic disease. These observations align with previous reports demonstrating that *miR-122* overexpression in hepatic stellate cells (HSCs) suppresses their activation and mitigates liver fibrosis [29]. Although our data confirm that hepatocytes are the predominant reservoir of miR-122, we also identified endogenous expression within HSCs. Consequently, the germline deletion of *miR-122* likely removes a critical regulatory brake, predisposing these cells to an exacerbated activated and pro-fibrotic phenotype.

The pro-regenerative effects of miR-122 loss observed in the healthy liver sharply contrasted with the response to chronic injury. In the fibrotic context, despite the presence of proliferating cells, albumin expression was drastically reduced, indicating impaired hepatocyte function. Importantly, the expansion of non-parenchymal cells and loss of hepatocyte function suggest a shift from effective parenchymal-driven regeneration to a maladaptive program dominated by hepatic stellate cell proliferation, inflammation and matrix deposition, processes previously linked to miR-122 dysregulation and hepatic stellate cell activation [29]. Although *miR-122^−/−^* fibrotic livers displayed strong Ki67 positivity, high Cyclin D1 and RhoA levels, and an apparently robust proliferative response, none of the mice survived the regeneration process after PHx. This complete lethality underscores a fundamental disconnect between proliferation and functional regeneration.

To rule out the specific influence of miR-122 deficiency from the confounding effects of pre-existing fibrosis, we performed pharmacological silencing of miR-122 in CCl_4_-treated WT mice with comparable baseline fibrosis levels. This approach resulted in significantly impaired fibrosis resolution following partial hepatectomy and a clear trend toward increased mortality, confirming that miR-122 deficiency directly contributes to hepatic dysfunction and reduced survival independent of initial fibrosis severity.

Proteomic analyses further support this interpretation. Even in the absence of fibrosis, *miR-122^−/−^* livers showed coordinated dysregulation of metabolic pathways, including early mitochondrial dysfunction, altered lipid processing, increased oxidative stress, and cytoskeletal remodeling. These results suggest that the loss of miR122 promotes a state of metabolic burnout. Even in non-fibrotic conditions, miR-122 deficiency induced bioenergetic alterations. In the fibrotic context, this escalated into a severe mitochondrial collapse, which aligns with prior evidence identifying miR-122 as a master regulator of the mitochondrial metabolic gene network, where its loss leads to impaired oxidative phosphorylation [30]. In this context, we propose that proliferating cells in *miR-122^−/−^* fibrotic livers cannot meet the high energy demands of division due to mitochondrial dysfunction compromising hepatocyte functions, like albumin synthesis, and leading to organ failure. This defines miR-122 as a critical growth regulator that ensures proliferation does not occur at the expense of metabolic viability.

Despite the evidence presented, certain limitations must be acknowledged. First, the use of a germline knockout model implies that miR-122 deficiency is present from embryogenesis. Although *miR-122^−/−^* mice are born healthy, they exhibit early metabolic priming that could distinguish their response from an acute, adult-onset loss of miR-122. Second, although proteomics revealed a clear signature of mitochondrial dysfunction, real-time functional assays were not performed to quantify the bioenergetic deficit ex vivo. Finally, this study focused on the consequences of miR-122 loss; future studies are needed to evaluate whether therapeutic reintroduction of miR-122 (e.g., via mimics) during the perioperative window can rescue the lethal phenotype.

In conclusion, our study demonstrates that miR-122 acts as a double-edged sword in liver regeneration. In the healthy liver, it acts as a brake to ensure orderly progression; in the fibrotic liver, it is an indispensable survival factor that maintains hepatocyte identity and mitochondrial function. The futile regeneration observed in its absence highlights miR-122 as a potential therapeutic target to prevent post-hepatectomy liver failure in patients with chronic liver disease.

## 4. Materials and Methods

### 4.1. Generation of miR-122 Knockout Mouse Model: miR-122 Knockout (miR-122^−/−^)

Mouse model was generated using the CRISPR/Cas9 system (PolyGene Transgenics, Rümlang, Zurich, Switzerland). Briefly, single guide RNAs (sgRNA) targeting the *miR-122* locus were designed, and three sgRNAs with high predicted specificity were selected: (1) 5′-GACTTTCCTTAGCAGAGCTG(TGG)-3′, (2) 5′-GATAATGGCGTTTGATGGTT(TGG)-3′ and (3) 5′-GTAGCTATTTAGTGTGATAA (TGG)-3′. These sgRNAs were injected into oocytes of C57BL/6J mice together with Cas9 protein, combining two of these sgRNAs in each injection. Embryos were implanted into pseudo-pregnant C57BL/6J females according to standard procedures and the pups were genotyped by PCR followed by sequence analysis. The positive founders were bred to establish the next generation, which was genotyped by PCR and DNA sequencing analysis. DNA sequencing revealed four different *miR-122*-deficient mouse clones: Mouse-E597.01 that was missing 45 bases in one strand, Mouse-E597.03 that was missing 8 bases in one strand + A insertion, Mouse-E597.18 that was an A insertion in one strand and Mouse-E597.21 that was missing 22 or 18 bases in both strands. Wild-type DNA was used as a negative control for sequencing in parallel. The mRNA transcribed from a targeted allele with frameshift undergoes nonsense-mediated decay (NMD). Experiments were conducted with *miR-122^−/−^* mice descended from the founder Mouse-E597.21 (Supplementary Figure S7), 50% male and 50% female. Age-matched wild-type 50% male and 50% female mice were included as controls. All animals were used at 8–12 weeks of age and housed under controlled temperature and humidity with a 12 h light/dark cycle and ad libitum access to standard pellet diet. We performed the study following the guidelines of the Investigation and Ethics Committee of Animal Experimentation of the University of Barcelona.

### 4.2. Experimental Model of Liver Fibrosis

All animal procedures were approved by the Investigation and Ethics Committee of Animal Experimentation of the University of Barcelona. Fibrosis was induced in 8-week-old male WT and *miR-122^−/−^* C57BL/6J mice (Jackson Laboratory, Bar Harbor, ME, USA) by intraperitoneal injection of CCl_4_ (1 mL CCl_4_/kg of body weight, previously diluted 1:8 *v*/*v* in corn oil) three times a week for 4 weeks. Age- and sex-matched mice without CCl_4_ treatment were used as control animals.

### 4.3. Patients

A retrospective cohort of patients treated at Hospital Clínic de Barcelona was analyzed. Informed consent was obtained from all patients, and the study was conducted in accordance with the principles of the Declaration of Helsinki and approved by the Institutional Review Board of Hospital Clínic de Barcelona (approval codes: HCB/12/7635 [R120316-069]; approved on 18 February 2020 and HCB/2023/0950; approved on 3 November 2023). Liver tissue samples were retrospectively collected from patients who underwent therapeutic partial hepatectomy, including individuals with and without cirrhosis (confirmed by biopsy in all cases). In total, eighteen liver tissues were analyzed, consisting of nine cirrhotic samples and nine control samples from healthy livers (non-cirrhotic samples). Cirrhotic tissues were obtained from patients with hepatitis C virus (HCV) infection. Control samples corresponded to histologically normal liver tissue collected during surgical removal of colorectal liver metastases, prior to vascular clamping. Any samples containing histological evidence of tumor tissue were excluded from the study.

### 4.4. Mouse Genotyping

Mouse genomic DNA was isolated from tail biopsies using a specific kit (Extract-N-Amp™ Tissue PCR Kit; Sigma-Aldrich, Darmstadt, Germany). PCR was performed using the primer pairs to amplify the *miR-122* gene (primer forward: 5′-AAGTCACGCGTGGAGTGG-3′ and primer reverse: 5′-AGTCCGTGTTCCCATAATTGGA-3′). PCR conditions were as follows: 30 cycles at 94 °C for 10 s, 57 °C for 100 s, and 72 °C for 15 s.

### 4.5. Sirius Red Staining

Collagen deposition was detected by Sirius Red staining. Hepatic tissue was fixed in a 4% solution of paraformaldehyde (pH = 7.4) and included in paraffin. Before the staining, paraffin-embedded liver sections (2 µm) were deparaffined and rehydrated. Tissue sections were stained in 0.1% Sirius Red F3B in saturated picric acid for 1 h and fixed in 1% acetic acid for 2 min. Finally, the slides were rinsed in distilled water, dehydrated, and mounted. Relative fibrotic area (expressed as a percentage of total liver area) was assessed in an optical microscope by analyzing 15 fields of Sirius Red-stained liver sections per animal. Each field was acquired at 100× magnification (Olympus BX51; Olympus Corporation, Shinjuku, Tokyo, Japan) and then analyzed using the morphometry software ImageJ (ImageJ version 1.52b; National Institutes of Health, Bethesda, MD, USA). To evaluate the relative fibrotic area, the collagen area measured was divided by the net field area and then multiplied by 100. Subtraction of vascular luminal area from the total field area yielded the final calculation of the net fibrotic area. The amount of fibrosis measured in each animal was analyzed, and the average value is presented as a percentage.

### 4.6. Two-Thirds Partial Hepatectomy

Partial hepatectomy was performed according to the technique described by Higgins and Anderson [31]. In brief, the abdomen was opened via a midline incision, and two thirds of the liver (median and left lobes) were removed. After PHx, WT mice were sacrificed at different time points, 1, 3, 6, 8 and 24 h, and the regenerating bottom right lobe was collected. For the comparative studies, WT mice and *miR-122^−/−^* mice were sacrificed at 2, 4 and 7 days. The percentage of liver regeneration was calculated following the formula of total weight of non-removed lobes/total body weight of mouse, and the volume of regenerated liver was measured by immersion method using Archimedes principle.

### 4.7. miR-122 Quantitative Expression

Total RNA was extracted from liver tissue from WT and CCl_4_-treated mice, human samples from control and cirrhotic patients, and isolated liver cells from WT and CCl_4_-treated mice using the Trizol reagent (Life Technologies, Carlsbad, CA, USA). RNA was reverse transcribed using Taqman microRNA assay Hsa-miR-122 (Thermo Fisher, Waltham, MA, USA) according to the manufacturer’s instructions. Subsequently, complementary DNA samples were amplified using the same assay (94 for 30 s, 55–60 °C for 30 s, and 72 °C for 1 min; 7900HT Fast Real-Time PCR System, Applied Biosystems, Waltham, MA, USA). To normalize the results, Taqman microRNA assay *RNU6B* was used as reference.

### 4.8. Immunofluorescence

Tissues were fixed in 4% paraformaldehyde, cryoprotected overnight in a 30% sucrose solution and embedded in optimal cutting temperature medium (Tissue-Tek^®^ O.C.T™ Compound, Sakura, Nagano, Japan). Next, frozen sections of 2 µm were rehydrated, blocked with 5% normal goat serum (NGS) and incubated overnight with rabbit anti-mouse Ki67 (Abcam, Cambridge, UK), rat anti-mouse endomucin (Abcam, Cambridge, UK), rabbit anti-mouse alpha smooth muscle actin (Abcam, Cambridge, UK) or goat anti-mouse albumin (Novus Biologicals, Abingdon, UK). Tissues for which immunostaining was performed without primary antibodies were used as negative controls. The binding sites of the primary antibodies were revealed with Alexa Fluor 488-conjugated donkey anti-rabbit IgG (Invitrogen, Carlsbad, CA, USA), Alexa Fluor 555-conjugated donkey anti-rabbit IgG (Invitrogen, Carlsbad, CA, USA), Alexa Fluor 488-conjugated rabbit anti-rat IgG (Invitrogen, Carlsbad, CA, USA) or Alexa Fluor 555-conjugated donkey anti-goat IgG (Invitrogen, Carlsbad, CA, USA). Cells were counterstained with 4,6-diamidino-2-phenylindole (DAPI) to visualize the nuclei. Stainings were analyzed using an Olympus BX51 microscope equipped with DP71 camera (Olympus Europa SE & CO.KG. Germany).

### 4.9. Western Blot Experiments

Cell lysates were prepared in a lysis buffer (Tris–HCl 20 mM pH = 7.4 containing 1% Triton X-100, 0.1% SDS, 50 mM NaCl, 2.5 mM EDTA, 1 mM Na_4_P_2_O_7_·10H_2_O, 20 mM NaF, 1 mM Na_3_VO_4_, 2 mM Pefabloc and Complete^®^ from Roche). Proteins were separated on a 10% SDS-polyacrylamide gel (Mini Protean III; Biorad, Hercules, CA, USA) and transferred for 2 h at 4 °C to nitrocellulose membranes (Transblot Transfer Medium; Biorad, Hercules, CA, USA). Then, the membranes were blocked and incubated at 4 °C with the following antibodies, rabbit anti-mouse Cyclin D1, mouse anti-mouse PCNA, rabbit anti-mouse CyclinG1, rabbit anti-mouse RhoA and rabbit anti-mouse β-actin HRP conjugate (Cell Signaling, Danvers, MA, USA), overnight in a 1:1000 dilution. Next, the membranes were incubated with goat anti-rabbit peroxidase-conjugated secondary antibody or goat anti-mouse peroxidase-conjugated secondary antibody at a 1:1000 dilution (Cell Signaling, Danvers, MA, USA) for 1 h at room temperature. The bands were visualized by chemiluminescence (Clarity Western ECLS substrate; Biorad, Hercules, CA, USA). The intensity of each band was quantified by ImageJ software (ImageJ version 1.52b; National Institutes of Health, Bethesda, MD, USA). Band intensities were measured and normalized to the indicated sample (shown as 1.00) on the same membrane. The molecular mass of the proteins was determined by comparing their electrophoretic mobility with that of the proteins contained in the Precision Plus Protein Standards Dual Color marker (Biorad, Hercules, CA, USA).

### 4.10. Hematoxylin and Eosin Staining

Frozen sections of 8 μm were rehydrated, and hepatic glycogen content was assessed in 8 μm liver sections that were embedded in paraffin and fixed in 10% buffered formaldehyde solution. Sections were periodic acid-Schiff stained according to manufacturer’s instructions (Sigma-Aldrich, Darmstadt, Germany), and counterstained with hematoxylin (Sigma-Aldrich, Darmstadt, Germany). Total hepatocyte cell area was quantified in hematoxylin and eosin-stained liver sections. The cell areas in the photomicrographs were measured using the software ImageJ (version 1.52b; National Institutes of Health, Bethesda, MD, USA). Stainings were analyzed using an Olympus BX51 microscope equipped with DP71 camera (Olympus Europa SE & CO.KG., Hamburg, Germany).

### 4.11. Quantification of Cellular and Nuclear Ploidy Profiles in Mouse Liver Sections

Nuclear ploidy was assessed using a method adapted from Bou-Nader et al. [32], and implemented in ImageJ (Rasband, WS, ImageJ, US National Institutes of Health, Bethesda, MD, USA, 1997–2016; https://imagej.net/ij/). Nuclei were stained with Hoechst and automatically detected. Nuclear area (in pixels)^2^ was measured as previously described [24,33,34], and hepatocyte ploidy was inferred from nuclear diameter, with only nuclei of circularity ≥ 0.7 included to exclude non-epithelial cells. A Gaussian mixture model was fit to the distribution of nuclear diameters, and thresholds were set to classify ploidy: 2n (30–60 pixels^2^), 4n (65–90 pixels^2^), and ≥8n (95–500 pixels^2^). Nuclei with areas < 30 pixels^2^ or >500 pixels^2^ were excluded. Each ploidy class was assigned a color code: 2n/purple, 4n/green, ≥8n/red. Cellular ploidy was quantified by counting mononuclear and binuclear hepatocytes in 10 randomly selected high-power fields per section (~5000 cells per sample).

### 4.12. Isolation of Hepatocytes and Hepatic Stellate Cells

Freshly isolated primary hepatocytes and hepatic stellate cells were obtained from the liver of the control and fibrotic mice. Cells were purified after collagenase A (Roche Diagnostics, Basel, Switzerland) retrograde perfusion and Nycodenz gradient (Sigma Chemical) as previously described [35,36].

### 4.13. miR-122 Inhibition Study

For miR-122 inhibition, CCl_4_-treated WT mice were treated with 1 mg/kg (i.v. tail vein) using scramble or anti-miR-122 (mmu-miR-122-5p *mir*Vana^TM^; Thermo Fisher, Waltham, MA, USA) 24 h prior the PHx. Mice were sacrificed at 2 and 7 days. For mice sacrificed on day 7, an extra administration of scramble or the anti-miR-122 was done 3 days after PHx.

### 4.14. Proteomic Sample Preparation and Mass Spectrometry Analysis

Protein extraction was performed by incubating the samples in a buffer containing 7 M urea, 2 M thiourea, and 4% CHAPS for 30 min at room temperature under agitation. Proteins were digested following a modified FASP protocol [37]. Trypsin was added in 50 mM ammonium bicarbonate at a trypsin:protein ratio of 1:10, and digestion was carried out overnight at 37 °C. Peptides were dried using an RVC2 25 speedvac concentrator (Christ, Osterode am Harz, Germany) and resuspended in 0.1% formic acid (FA). Prior to analysis, peptides were desalted using C18 stage tips (Sigma-Aldrich, Darmstadt, Germany) and resuspended in 0.1% FA. Mass spectrometry analysis was performed using a timsTOF Pro with PASEF (Bruker Daltonics, Billerica, MA, USA) coupled online to an Evosep ONE liquid chromatograph (Evosep, Odense, Denmark). A total of 200 ng of peptides were directly loaded onto the Evosep ONE and separated using the 15 samples-per-day protocol. DIA data was analyzed using DIA-NN software (version 1.8.1) for protein identification and quantification with default parameters. Searches were conducted against a Mus musculus protein database from UniProt in library-free mode. Carbamidomethylation of cysteines was set as a fixed modification, while oxidation of methionines was considered a variable modification. Data was processed in Perseus (log2 transformation, filtering, and imputation), and statistical analysis was performed using a two-sided Student’s *t*-test with FDR correction.

### 4.15. Bioinformatic Analysis

Differential protein abundance between wild-type and *miR-122^−/−^* mice, in both healthy and fibrotic conditions, was assessed using a two-sided Student’s *t*-test, with *p*-values corrected for multiple testing using the Benjamini–Hochberg false discovery rate (FDR). Proteins with an FDR-adjusted *p*-value (*q* value) < 0.05 were considered statistically significant. For each protein, log2 mean expression values were calculated by averaging across biological replicates, and log2 fold change (log2FC) was defined as *miR-122^−/−^* minus WT. Protein identifiers were annotated using the UniProtKB database. Data visualization and additional analyses were performed using R (version 4.4.2). Functional enrichment and pathway analysis were performed using Ingenuity Pathway Analysis (IPA, Qiagen, ver. 2025-11). Proteins with an adjusted *q* value < 0.05 and |log2FC| ≥ 0.5 were included in the analysis. Canonical pathways, upstream regulators, and network interactions were identified using the default settings in IPA.

### 4.16. Statistical Analysis

Quantitative data were analyzed using GraphPad Prism 5 (GraphPad Software, Inc, San Diego, CA, USA), and statistical analysis of the results was analyzed using unpaired Student’s *t* test and ANOVA models (with Tukey’s post hoc test) with normally distributed data or small animal datasets (*n* < 5). Partial hepatectomy mortality scores were analyzed by log-rank test and survival curves were generated using the product limit method of Kaplan and Meier. For the patient data, the Mann–Whitney U-test was used. Differences were considered to be significant at a *p*-value < 0.05. The data are presented as the mean ± standard error of the mean, if not specified otherwise.

## Data Availability

All data generated or analyzed during this study are included in this published article and its Supplementary Information Files.

## Supplementary Materials

The following supporting information can be downloaded at https://www.mdpi.com/article/10.3390/ijms27073149/s1.

## References

[1] Michalopoulos G.K. (2017). Hepatostat: Liver Regeneration and Normal Liver Tissue Maintenance. Hepatology.

[2] Campana L., Esser H., Huch M., Forbes S. (2021). Liver Regeneration and Inflammation: From Fundamental Science to Clinical Applications. Nat. Rev. Mol. Cell Biol..

[3] Maier E., Stättner S., Carrion-Alvarez L., Di Martino M., Olthof P., Primavesi F., Sochorova D., Van Laarhoven S., Balakrishnan A., Breitkopf R. (2025). The E-AHPBA—ESSO—Innsbruck Consensus Recommendations on Peri- and Postoperative Management Following Liver Resection. Br. J. Surg..

[4] Forbes S.J., Newsome P.N. (2016). Liver Regeneration—Mechanisms and Models to Clinical Application. Nat. Rev. Gastroenterol. Hepatol..

[5] Bandiera S., Pfeffer S., Baumert T.F., Zeisel M.B. (2015). miR-122—A Key Factor and Therapeutic Target in Liver Disease. J. Hepatol..

[6] Szabo G., Bala S. (2013). MicroRNAs in Liver Disease. Nat. Rev. Gastroenterol. Hepatol..

[7] Kozlov D.S., Rodimova S.A., Kuznetsova D.S. (2023). The Role of MicroRNAs in Liver Functioning: From Biogenesis to Therapeutic Approaches (Review). Sovrem. Teh. Med..

[8] Hu J., Xu Y., Hao J., Wang S., Li C., Meng S. (2012). MiR-122 in Hepatic Function and Liver Diseases. Protein Cell.

[9] Girard M., Jacquemin E., Munnich A., Lyonnet S., Henrion-Caude A. (2008). miR-122, a Paradigm for the Role of microRNAs in the Liver. J. Hepatol..

[10] Wen J., Friedman J.R. (2012). miR-122 Regulates Hepatic Lipid Metabolism and Tumor Suppression. J. Clin. Investig..

[11] Bai S., Nasser M.W., Wang B., Hsu S.-H., Datta J., Kutay H., Yadav A., Nuovo G., Kumar P., Ghoshal K. (2009). MicroRNA-122 Inhibits Tumorigenic Properties of Hepatocellular Carcinoma Cells and Sensitizes These Cells to Sorafenib. J. Biol. Chem..

[12] Nakao K., Miyaaki H., Ichikawa T. (2014). Antitumor Function of microRNA-122 against Hepatocellular Carcinoma. J. Gastroenterol..

[13] Hsu S., Wang B., Kota J., Yu J., Costinean S., Kutay H., Yu L., Bai S., La Perle K., Chivukula R.R. (2012). Essential Metabolic, Anti-Inflammatory, and Anti-Tumorigenic Functions of miR-122 in Liver. J. Clin. Investig..

[14] Coulouarn C., Factor V.M., Andersen J.B., Durkin M.E., Thorgeirsson S.S. (2009). Loss of miR-122 Expression in Liver Cancer Correlates with Suppression of the Hepatic Phenotype and Gain of Metastatic Properties. Oncogene.

[15] Tsai W.-C., Hsu S.-D., Hsu C.-S., Lai T.-C., Chen S.-J., Shen R., Huang Y., Chen H.-C., Lee C.-H., Tsai T.-F. (2012). MicroRNA-122 Plays a Critical Role in Liver Homeostasis and Hepatocarcinogenesis. J. Clin. Investig..

[16] Song G., Sharma A.D., Roll G.R., Ng R., Lee A.Y., Blelloch R.H., Frandsen N.M., Willenbring H. (2010). Micrornas Control Hepatocyte Proliferation During Liver Regeneration. Hepatology.

[17] Halász T. (2015). miR-122 Negatively Correlates with Liver Fibrosis as Detected by Histology and FibroScan. WJG.

[18] Gramantieri L., Ferracin M., Fornari F., Veronese A., Sabbioni S., Liu C.-G., Calin G.A., Giovannini C., Ferrazzi E., Grazi G.L. (2007). Cyclin G1 Is a Target of miR-122a, a MicroRNA Frequently Down-Regulated in Human Hepatocellular Carcinoma. Cancer Res..

[19] Hsu S., Delgado E.R., Otero P.A., Teng K., Kutay H., Meehan K.M., Moroney J.B., Monga J.K., Hand N.J., Friedman J.R. (2016). MicroRNA-122 Regulates Polyploidization in the Murine Liver. Hepatology.

[20] Margall-Ducos G., Celton-Morizur S., Couton D., Brégerie O., Desdouets C. (2007). Liver Tetraploidization Is Controlled by a New Process of Incomplete Cytokinesis. J. Cell Sci..

[21] Welsh C.F., Roovers K., Villanueva J., Liu Y., Schwartz M.A., Assoian R.K. (2001). Timing of Cyclin D1 Expression within G1 Phase Is Controlled by Rho. Nat. Cell Biol..

[22] Chun K.-H. (2022). Molecular Targets and Signaling Pathways of microRNA-122 in Hepatocellular Carcinoma. Pharmaceutics.

[23] Ma L., Liu J., Shen J., Liu L., Wu J., Li W., Luo J., Chen Q., Qian C. (2010). Expression of miR-122 Mediated by Adenoviral Vector Induces Apoptosis and Cell Cycle Arrest of Cancer Cells. Cancer Biol. Ther..

[24] Gentric G., Maillet V., Paradis V., Couton D., L’Hermitte A., Panasyuk G., Fromenty B., Celton-Morizur S., Desdouets C. (2015). Oxidative Stress Promotes Pathologic Polyploidization in Nonalcoholic Fatty Liver Disease. J. Clin. Investig..

[25] Perramón M., Carvajal S., Reichenbach V., Fernández-Varo G., Boix L., Macias-Muñoz L., Melgar-Lesmes P., Bruix J., Melmed S., Lamas S. (2022). The Pituitary Tumour-transforming Gene 1/Delta-like Homologue 1 Pathway Plays a Key Role in Liver Fibrogenesis. Liver Int..

[26] Ribera J., Pauta M., Melgar-Lesmes P., Córdoba B., Bosch A., Calvo M., Rodrigo-Torres D., Sancho-Bru P., Mira A., Jiménez W. (2017). A Small Population of Liver Endothelial Cells Undergoes Endothelial-to-Mesenchymal Transition in Response to Chronic Liver Injury. Am. J. Physiol. Gastrointest. Liver Physiol..

[27] Morán-Salvador E., Titos E., Rius B., González-Périz A., García-Alonso V., López-Vicario C., Miquel R., Barak Y., Arroyo V., Clària J. (2013). Cell-Specific PPARγ Deficiency Establishes Anti-Inflammatory and Anti-Fibrogenic Properties for This Nuclear Receptor in Non-Parenchymal Liver Cells. J. Hepatol..

[28] Fujii T., Fuchs B.C., Yamada S., Lauwers G.Y., Kulu Y., Goodwin J.M., Lanuti M., Tanabe K.K. (2010). Mouse Model of Carbon Tetrachloride Induced Liver Fibrosis: Histopathological Changes and Expression of CD133 and Epidermal Growth Factor. BMC Gastroenterol..

[29] Ma J., Zhao Q., Chen M., Wang W., He B., Jiang Y., Li Y. (2022). microRNA-122 Inhibits Hepatic Stellate Cell Proliferation and Activation in Vitro and Represses Carbon Tetrachloride-Induced Liver Cirrhosis in Mice. Ann. Hepatol..

[30] Burchard J., Zhang C., Liu A.M., Poon R.T.P., Lee N.P.Y., Wong K., Sham P.C., Lam B.Y., Ferguson M.D., Tokiwa G. (2010). microRNA-122 as a Regulator of Mitochondrial Metabolic Gene Network in Hepatocellular Carcinoma. Mol. Syst. Biol..

[31] Higgins G.M., Anderson R.M. (1931). Experimental Pathology of Liver: Restoration of Liver of White Rat Following Partial Surgical Removal. Arch. Pathol. Lab Med..

[32] Bou-Nader M., Caruso S., Donne R., Celton-Morizur S., Calderaro J., Gentric G., Cadoux M., L’Hermitte A., Klein C., Guilbert T. (2020). Polyploidy Spectrum: A New Marker in HCC Classification. Gut.

[33] Miyaoka Y., Ebato K., Kato H., Arakawa S., Shimizu S., Miyajima A. (2012). Hypertrophy and Unconventional Cell Division of Hepatocytes Underlie Liver Regeneration. Curr. Biol..

[34] Tanami S., Ben-Moshe S., Elkayam A., Mayo A., Bahar Halpern K., Itzkovitz S. (2017). Dynamic Zonation of Liver Polyploidy. Cell Tissue Res..

[35] Morales-ruiz M., Cejudo-martín P., Fernández-varo G., Tugues S., Ros J., Angeli P., Rivera F., Arroyo V., Rodés J., Sessa W.C. (2003). Transduction of the Liver with Activated Akt Normalizes Portal Pressure in Cirrhotic Rats. Gastroenterology.

[36] Tugues S., Morales–Ruiz M., Fernandez–Varo G., Ros J., Arteta D., Muñoz–Luque J., Arroyo V., Rodés J., Jiménez W. (2005). Microarray Analysis of Endothelial Differentially Expressed Genes in Liver of Cirrhotic Rats. Gastroenterology.

[37] Wiśniewski J.R., Zougman A., Nagaraj N., Mann M. (2009). Universal Sample Preparation Method for Proteome Analysis. Nat. Methods.

