# Supplementary material for: miR-122 Deficiency in Mice Enhances Regeneration in Healthy Liver but Drives Pathological Repair and Functional Decline in Fibrotic Liver
Source: Int J Mol Sci. 2026 Mar 30;27(7):3149. doi: 10.3390/ijms27073149 (PMC13072873; doi:10.3390/ijms27073149)
Supplement: Supplementary file 1 [file ijms-27-03149-s001.zip › ijms-4199780-supplementary materials.pdf]

Supplementary file

miR-122 Deficiency in mice Enhances Regeneration in Healthy Liver but Drives Pathological Repair and Functional Decline in Fibrotic Liver

Jordi Ribera[1*], Anna Cardona-Simó[1,2*], Irene Portolés[3,4], Esther Samper[5], Loreto Boix[5], Aleix B. Fabregat-Bolufer[1], Esther Fernández-Galán[1], María Rodríguez-Garcia[1], Mikel Azkargorta[6], Felix Elortza[6], Séverine Celton-Morizur[3,4], Chantal Desdouets[3,4], Pedro Melgar-Lesmes[1,2], Wladimiro Jiménez[1,2], Gregori Casals[1,7], Manuel Morales-Ruiz[1,2&]

[1]Biochemistry and Molecular Genetics Department, Hospital Clínic of Barcelona, Institut d'Investigacions Biomèdiques August Pi i Sunyer (IDIBAPS), Centro de Investigación Biomédica en Red de Enfermedades Hepáticas y Digestivas (CIBERehd), 08036 Barcelona, Spain. [2]Biomedicine Department, Faculty of Medicine and Health Sciences, University of Barcelona, 08036 Barcelona, Spain. [3]Centre de Recherche des Cordeliers, Sorbonne Université, INSERM, Université de Paris, F-75006 Paris, France. [4]Genomic Instability, Metabolism, Immunity and Liver Tumorigenesis laboratory, Equipe labellisée par la Ligue Nationale Contre le Cancer, F-75006 Paris, France. [5]Barcelona Clinic Liver Cancer Group, Liver Unit, Hospital Clinic, University of Barcelona, IDIBAPS, CIBERehd, 08036 Barcelona, Spain. [6]Proteomics Platform, CIC bioGUNE, Basque Research and Technology Alliance (BRTA), CIBERehd, 48160 Derio, Spain. [7]Department of fundamental and clinical nursing, Faculty of Nursing, University of Barcelona, Spain.

Table of content:

1. Additional Figures and Legends

ADDITIONAL FIGURES AND LEGENDS

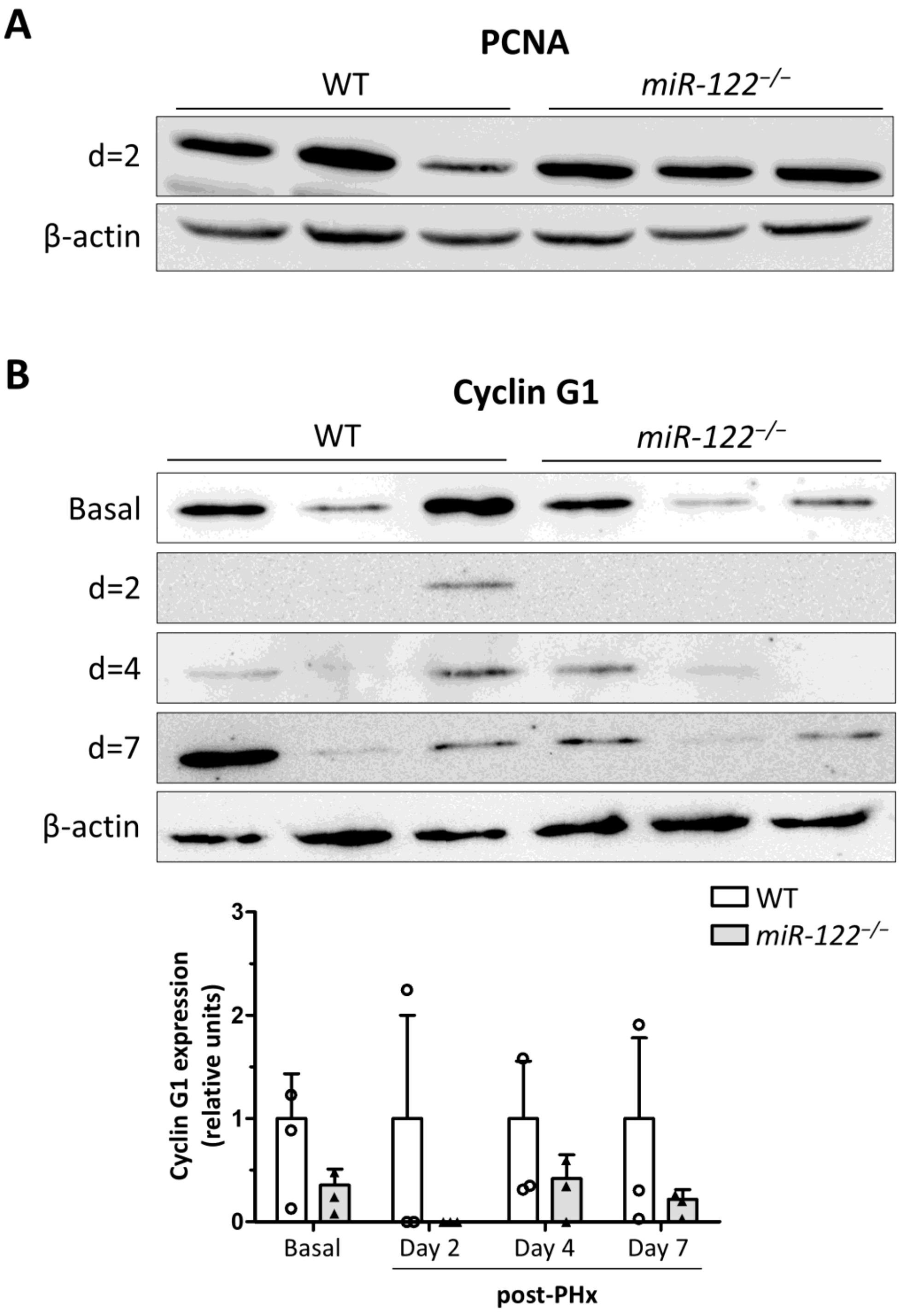

Supplementary Figure S1. Lack of *miR-122* does not affect the expression of PCNA nor Cyclin G1 during liver regeneration. (**A**) The expression of PCNA

protein was evaluated by Western blot using liver tissue lysates from wild-type (WT) and *miR-122* knockout (*miR-122*$^{-/-}$) mice 2 days after partial hepatectomy (PHx). β-actin was used as a loading control (*n* = 3). (**B**) The expression of Cyclin G1 protein was evaluated by Western blot using liver tissue lysates from WT and *miR-122*$^{-/-}$ mice at different points after PHx. β-actin was used as a loading control. The densitometric analysis of the protein expression is shown on the bottom graph. Bars represent the mean ± SEM (*n* = 3 for each time point).

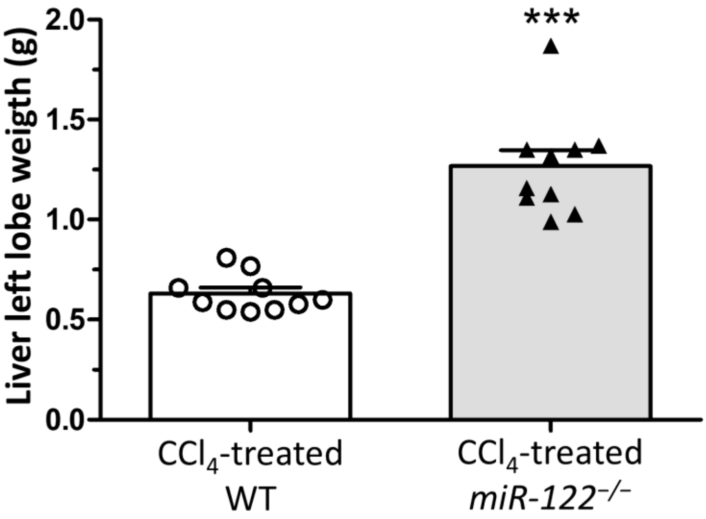

Supplementary Figure S2. Liver size is significantly increased in the *miR-122*$^{-/-}$ mouse after CCl_4 treatment. Graph showing the liver left lobe weight from CCl_4-treated wild-type (WT) and CCl_4-treated *miR-122* knockout (*miR-122*$^{-/-}$) mice extracted during the procedure of partial hepatectomy. Bars represent the mean ± SEM; ***p=0.0001 *vs.* CCl_4-treated WT mice (*n* = 10, 5 males and 5 females).

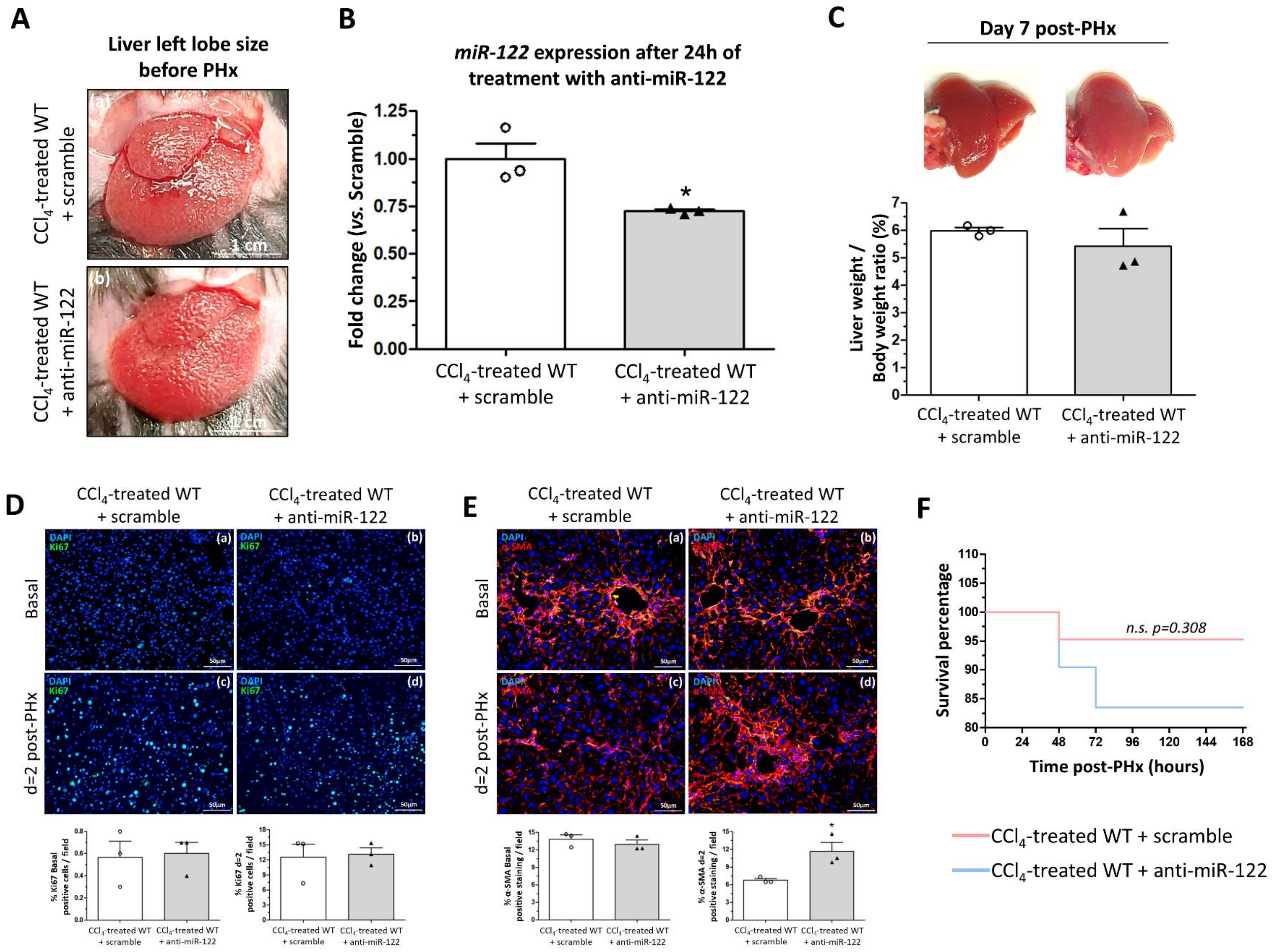

Supplementary Figure S3. Silencing of miR-122 recapitulates impaired liver function and reduced survival in fibrotic wild-type mice. (**A**) Representative images of the liver left lobe from a CCl_4-treated wild-type (WT) mouse injected with scramble and a CCl_4-treated WT mouse injected with the anti-miR-122 before partial hepatectomy. (**B**) Liver tissue from CCl_4-treated WT mice injected with scramble and a CCl_4-treated WT mice injected with the anti-miR-122 (n = 3) was lysed in trizol and total mRNA was extracted. *miR-122* expression was analyzed by RT-qPCR using Taqman microRNA assay Hsa-miR-122, as

described in Methods. Graph shows the expression levels for *miR-122* as fold change relative to *RNU6B* levels. Bars represent the mean ± SEM; *p=0.029 *vs.* CCl_4-treated WT mouse injected with scramble. (**C**) Hepatic regenerative index (liver weight/body weight ratio) obtained in CCl_4-treated WT mice injected with scramble and a CCl_4-treated WT mice injected with the anti-miR-122 seven days after PHx. Bars represent the mean ± SEM (n = 3, 1 male and 2 females). (**D**) Representative merged images of immunofluorescence staining of Ki67-positive cells (green) and DAPI (blue) in CCl_4-treated WT mice injected with scramble and CCl_4-treated WT mice injected with the anti-miR-122 livers at basal (panels a and b) and 2 days (panels c and d) following partial hepatectomy (PHx). Original magnification ×200. Lower graphs show the computer-assisted quantification of Ki67-positive cells/total nuclei. Bars represent mean ± SEM (n = 3, 2 males and 1 female). (**E**) α-smooth muscle actin (α-SMA) immunostaining of activated hepatic stellate cells in CCl_4-treated WT mice injected with scramble and CCl_4-treated WT mice injected with the anti-miR-122 at basal (panels a and b) and 2 days (panels c and d) following PHx. Original magnification: 200×. Quantification of the percentage of α-SMA positive staining is shown in the lower graphs. Bars represent the mean ± SEM; *p=0.033 *vs.* CCl_4-treated WT mice injected with scramble (n = 3, 2 males and 1 female). (**F**) Survival graph showing CCl_4-treated WT mice injected with scramble and CCl_4-treated WT mice injected with the anti-miR-122 mortality from 24 hours to 7 days after PHx (n = 8, 4 males and 4 females).

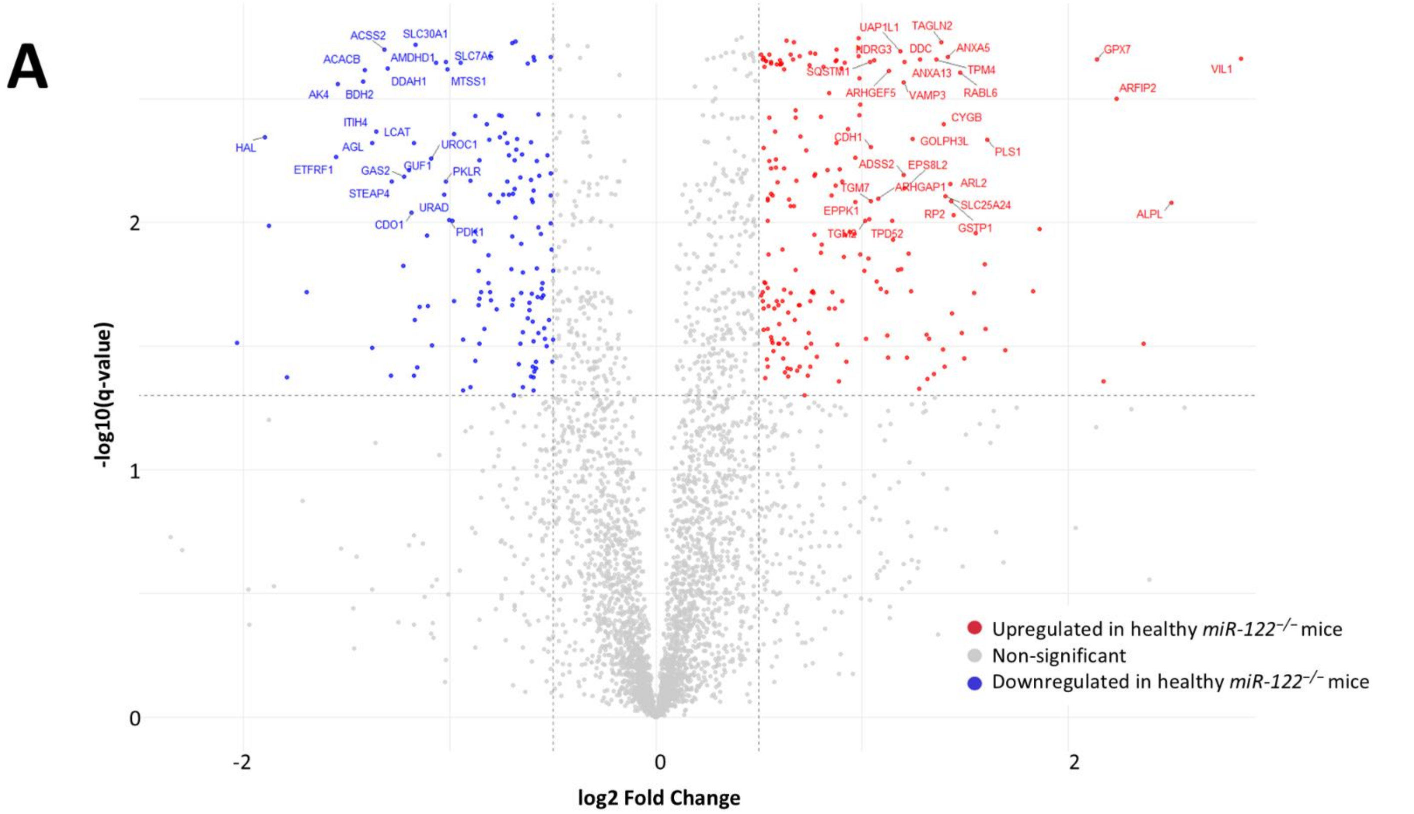

Wild-type mouse *vs.* *miR-122*$^{-/-}$ mouse 2 days after PHx (liver tissue)

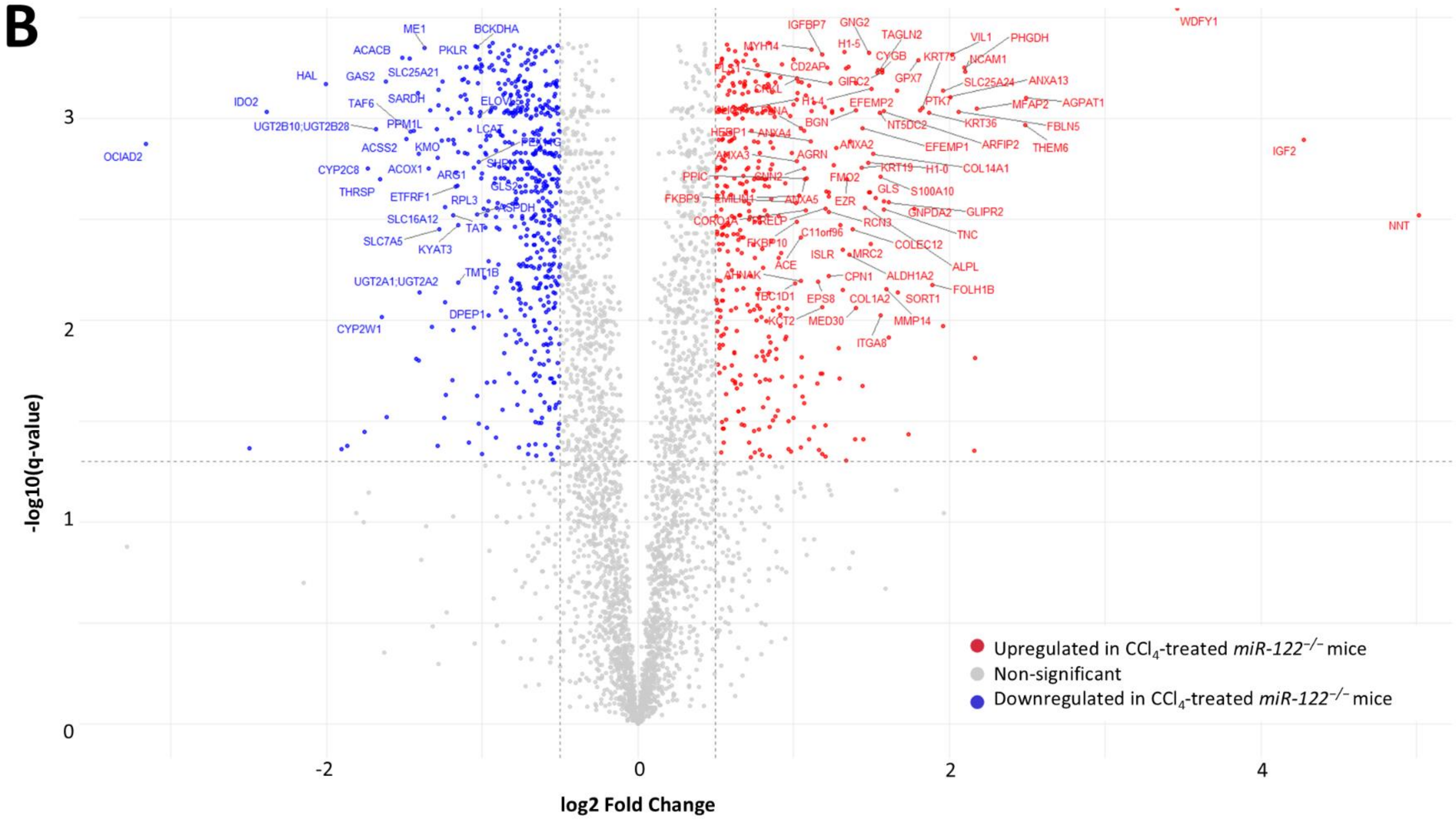

CCl_4-treated wild-type mouse *vs.* CCl_4-treated *miR-122*$^{-/-}$ mouse 2 days after PHx (liver tissue)

Supplementary Figure S4. Overall distribution of differentially expressed hepatic proteins in the *miR-122* knockout mice during liver regeneration. (**A-B**) The differentially expressed liver proteins analyzed by volcano plots between wild-type mouse (WT) and *miR-122* knockout (*miR-122*$^{-/-}$) mouse (upper volcano plot, **A**), and between CCl_4-treated WT mouse and CCl_4-treated *miR-122*$^{-/-}$ mouse (lower volcano plot, **B**), 2 days after partial hepatectomy. Proteins with higher expression in *miR-122*$^{-/-}$ mice are shown in red, and proteins with lower expression in *miR-122*$^{-/-}$ are shown in blue. Proteins were considered significantly differentially expressed if q-value<0.05 and |Log2FC|≥0.5. Proteins with q< 0.01 and |Log2FC|≥1 are labeled with their names (n = 5).

A

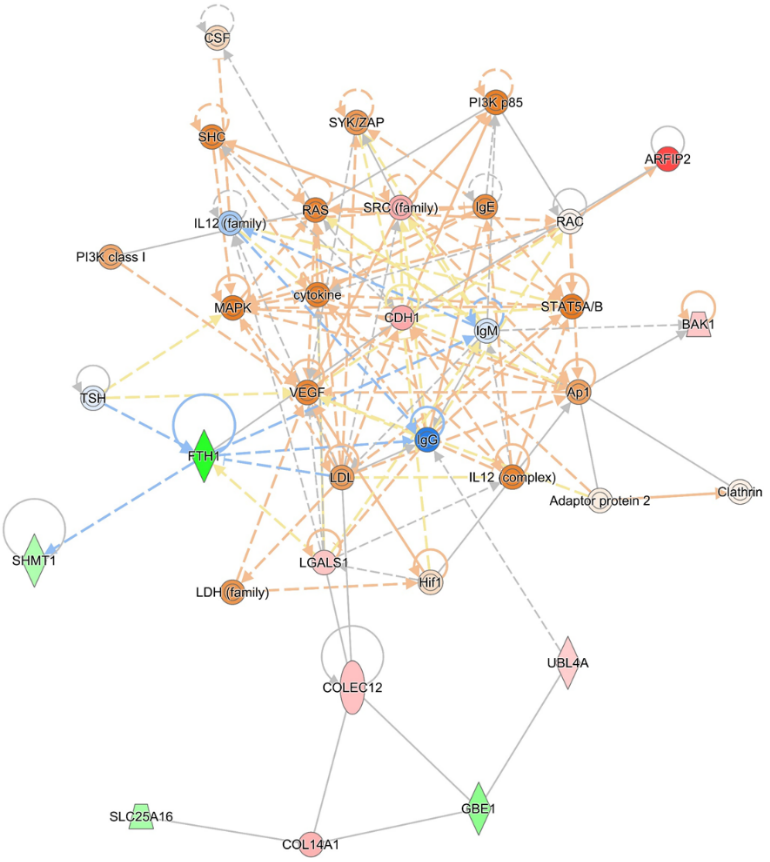

Network 2. Cell death and survival, and tissue morphology*; Score 20.*

B

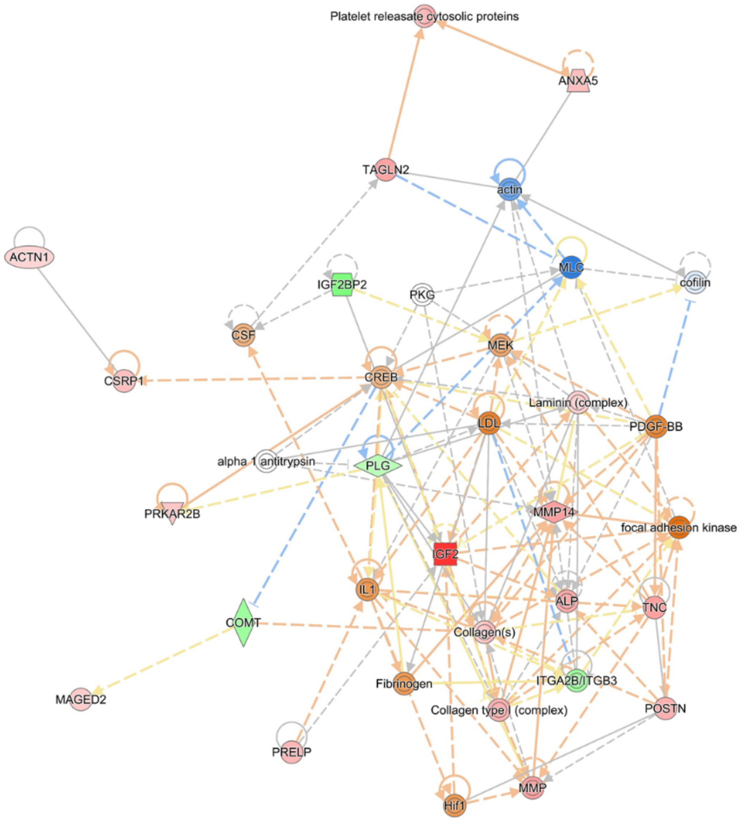

Network 2. Connective tissue development and function, embryonic development and organ morphology*; Score 18.*

Supplementary Figure S5. Network analysis of hepatic molecular changes derived from *miR-122* deficiency during liver regeneration. (**A-B**) Networks of pathways obtained from the proteomic analysis of wild-type (WT) and *miR-122* knockout (*miR-122*$^{-/-}$) livers (upper network, **A**) and CCl_4-treated WT and the CCl_4-treated *miR-122*$^{-/-}$ livers (lower network, **B**), 2 days after partial hepatectomy were algorithmically generated based on their connectivity. The proteins are represented as nodes, and the biological relationship between two nodes is represented as an edge (line). A colored node indicates a protein that was detected by the proteomic screening (red: overexpressed, green: reduced expression, orange: predicted activation and blue: predicted inhibition in *miR-122*$^{-/-}$ liver). Intensity of the color indicates the magnitude of expression change or the confidence of the prediction. Nodes are displayed using various shapes that represent the functional class of the proteins. Edges with dashed lines show indirect interaction (n = 5).

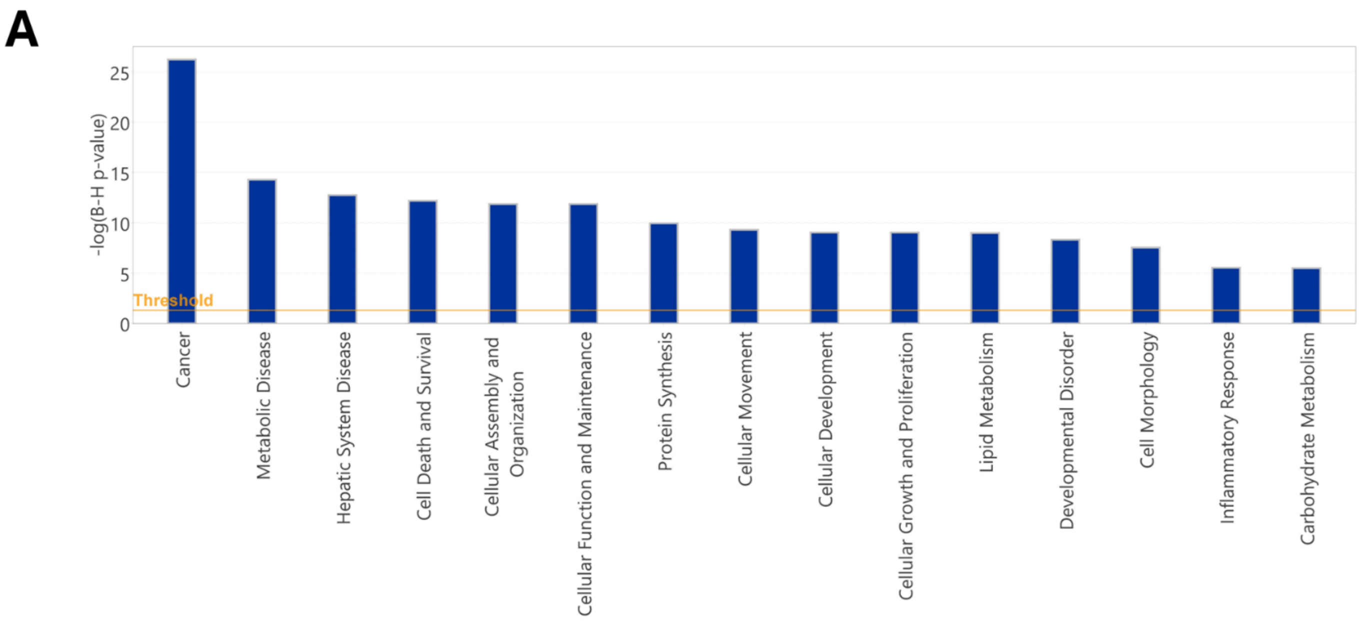

1. IPA diseases and function analysis in healthy context

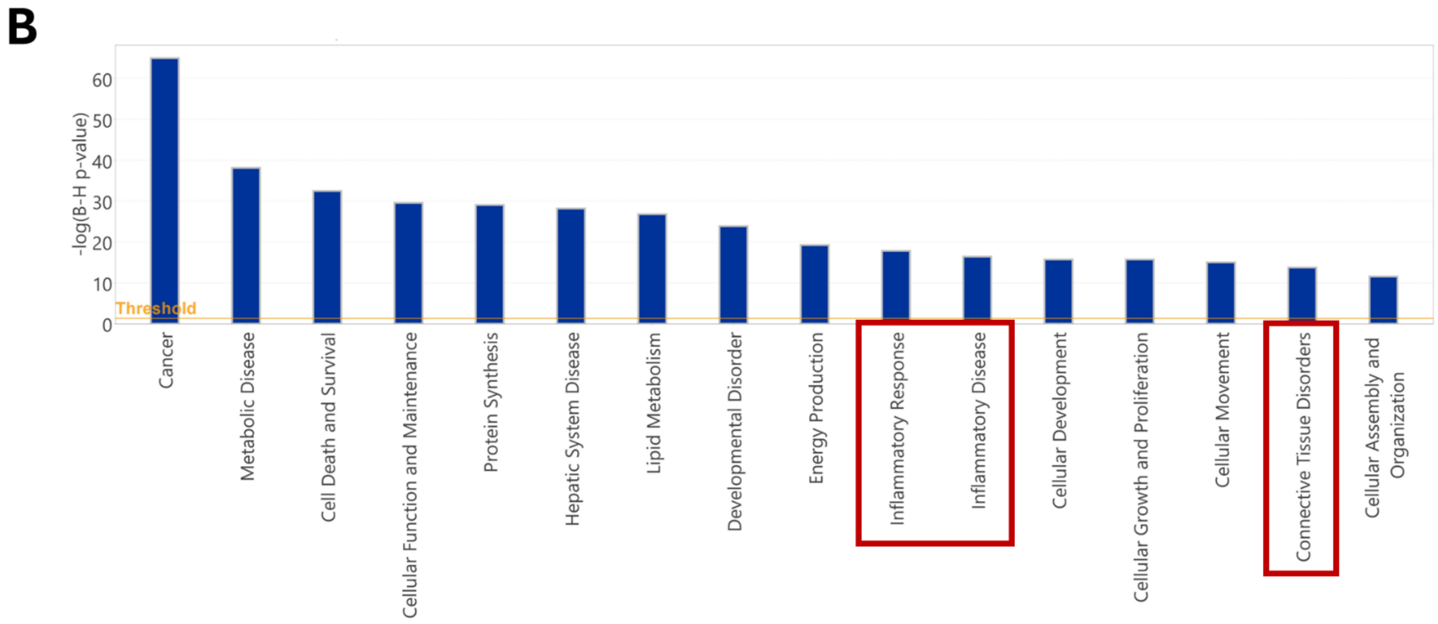

2. IPA diseases and function analysis in fibrotic context

Supplementary Figure S6. Bioinformatic analysis of the proteome obtained from *miR-122* knockout mice in both healthy and fibrotic context. (**A-B**) The

comparisons of diseases and biological functions affected by the knockout of *miR-122* activity in the liver were modeled using the Ingenuity Pathways Knowledge Base (IPA). The results obtained were compared with global molecular networks using the Fisher's Exact Test. The resulting *P*-values were adjusted for multiple comparisons using the Benjamini-Hochberg method to control the false discovery rate. After multi-test adjustment, differences were considered as significant at a *P* value less than 0.05. The orange line across the graph indicates the point where the significance value equals 0.05. In the upper graph (**A**), IPA diseases and function analysis in a healthy context, and in the lower graph (**B**), IPA diseases and function analysis in a fibrotic context (the red boxes indicate the differential processes activated in the fibrotic in comparison to healthy context) ($n = 5$).

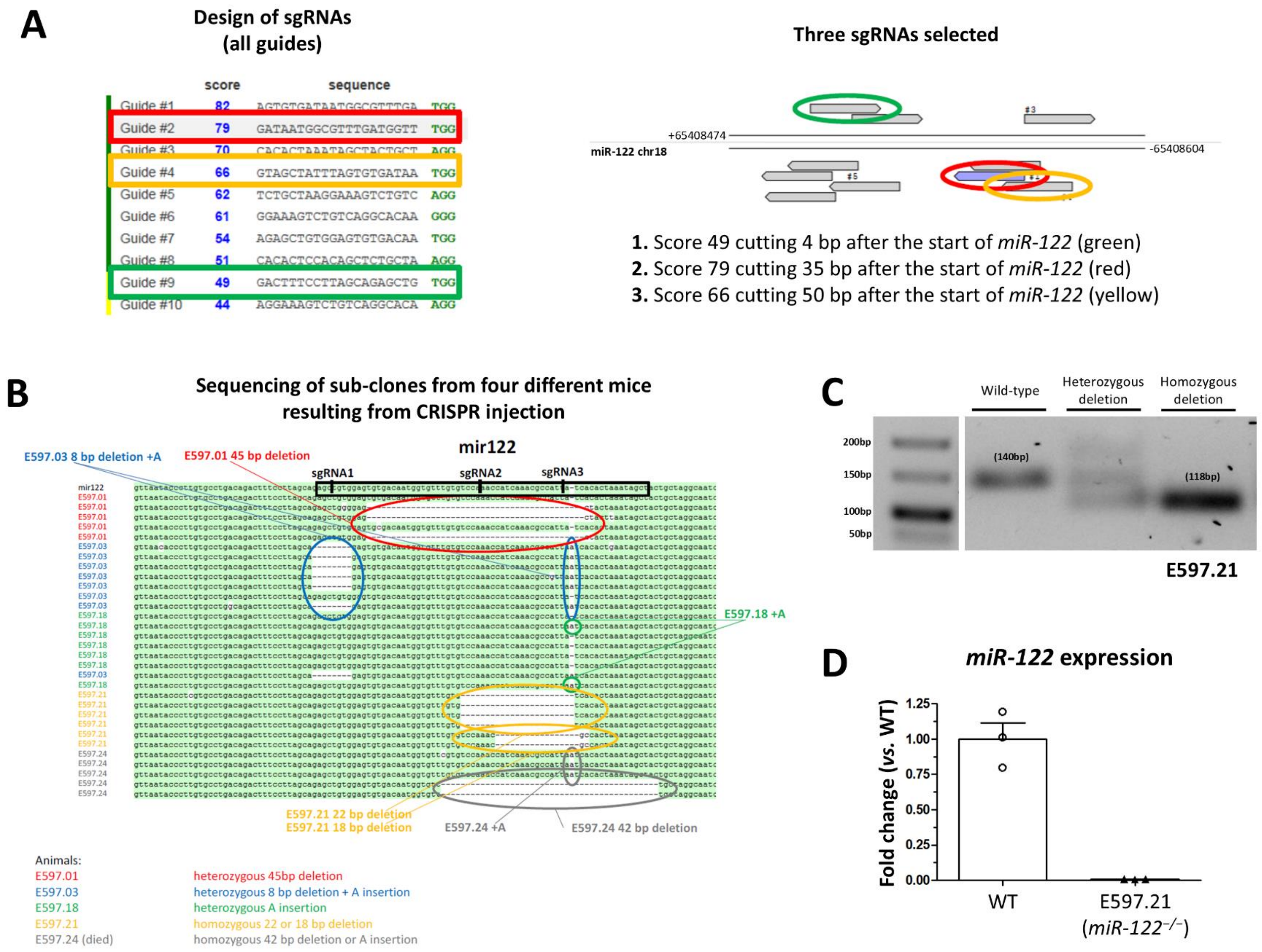

Supplementary Figure S7. *miR-122* knockout mouse model. (**A**) Different guide RNAs for the *miR-122* gene were generated using bioinformatic tools for the miR-122 locus. The first selected sgRNA has a score of 49 (green), the second of 66 (yellow) and the third score is 79 (red). (**B**) The reference sequence for the miR122 locus is shown in black with the three different cutting sites for the sgRNAs (black bars). About 6 clones were analyzed from each mouse (indicated in different colors before the sequence). The resulting mutations in the different sub-clones and the corresponding mice are highlighted. (**C**) For mice genotyping, PCR was performed to amplify specific

DNA bands for wild-type and knockout genotype. The following primers were used in the same PCR mix for each genomic DNA sample: Forward: 5'-AAGTCACGCGTGGAGTGG-3' and Reverse: 5'-AGTCCGTGTTCCCATAATTGGA-3' to amplify a band of 140bp specific for wild-type genotype and a band of 118bp specific for knockout genotype. The number of observed bands in each lane and their position in the agarose gel depending on their length were used to determine the genotype of every animal: a unique band of 140bp corresponded to *miR-122*$^{+/+}$ mice, a unique band of 118bp corresponded to *miR-122*$^{-/-}$ mice and the presence of both bands in the same lane corresponded to *miR-122*$^{+/-}$ mice. (**D**) Liver tissue from wild-type mice and *miR-122*$^{-/-}$ mice was lysed in trizol and total mRNA was extracted. *miR-122* expression was analyzed by RT-qPCR using Taqman microRNA assay Hsa-miR-122, as described in Methods. Graph shows the expression levels for *miR-122* as fold change relative to *Rnu6b* levels. Bars represent the mean ± SEM (*n* = 3).
