# Supplementary figures and images for: miR-122 Deficiency in Mice Enhances Regeneration in Healthy Liver but Drives Pathological Repair and Functional Decline in Fibrotic Liver
Source: Int J Mol Sci. 2026 Mar 30;27(7):3149. doi: 10.3390/ijms27073149 (PMC13072873; doi:10.3390/ijms27073149)

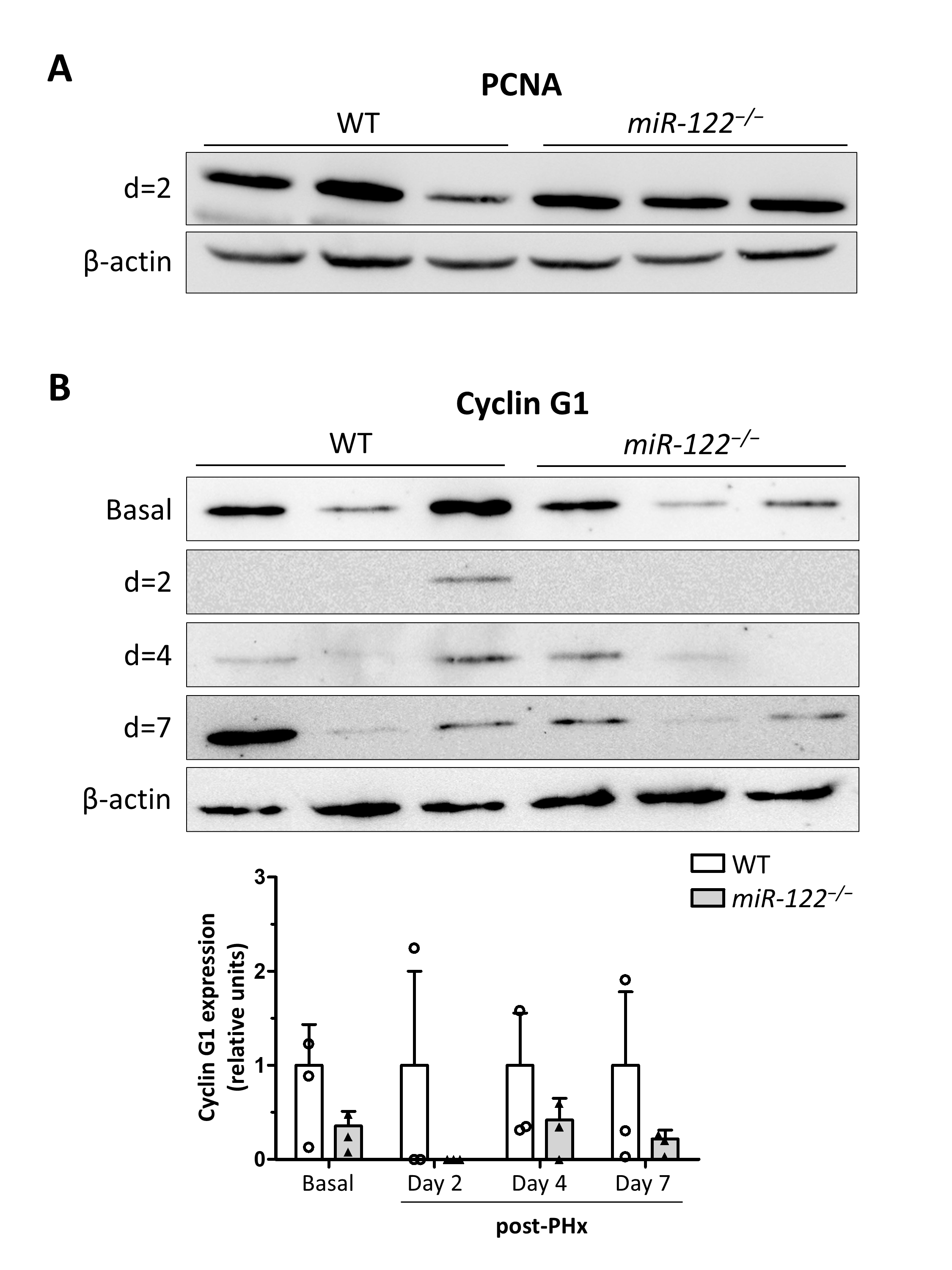

Supplement: Supplementary file 1 [file ijms-27-03149-s001.zip › Supplementari Figure S1 png.PNG]

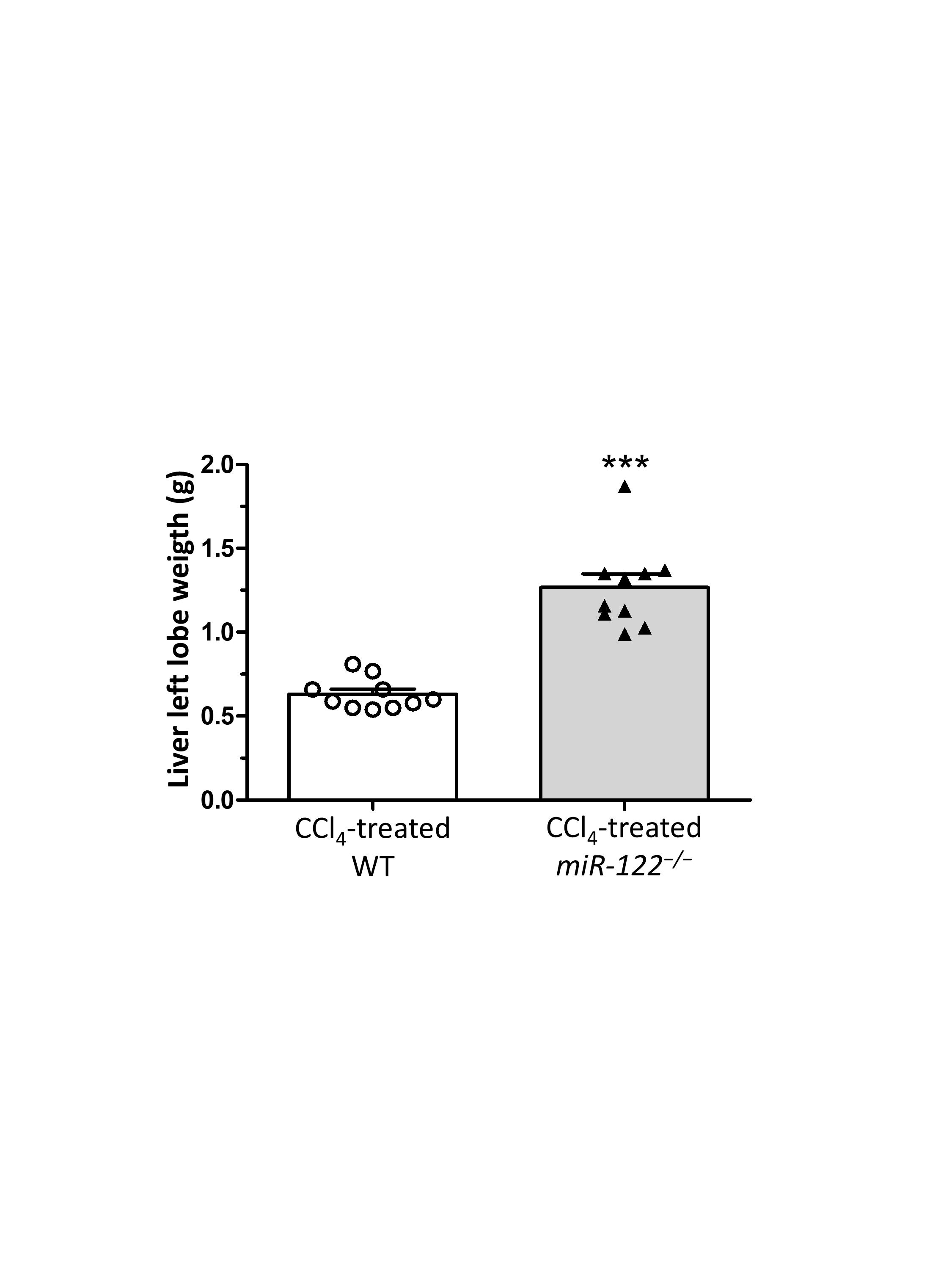

Supplement: Supplementary file 1 [file ijms-27-03149-s001.zip › Supplementari Figure S2 png.PNG]

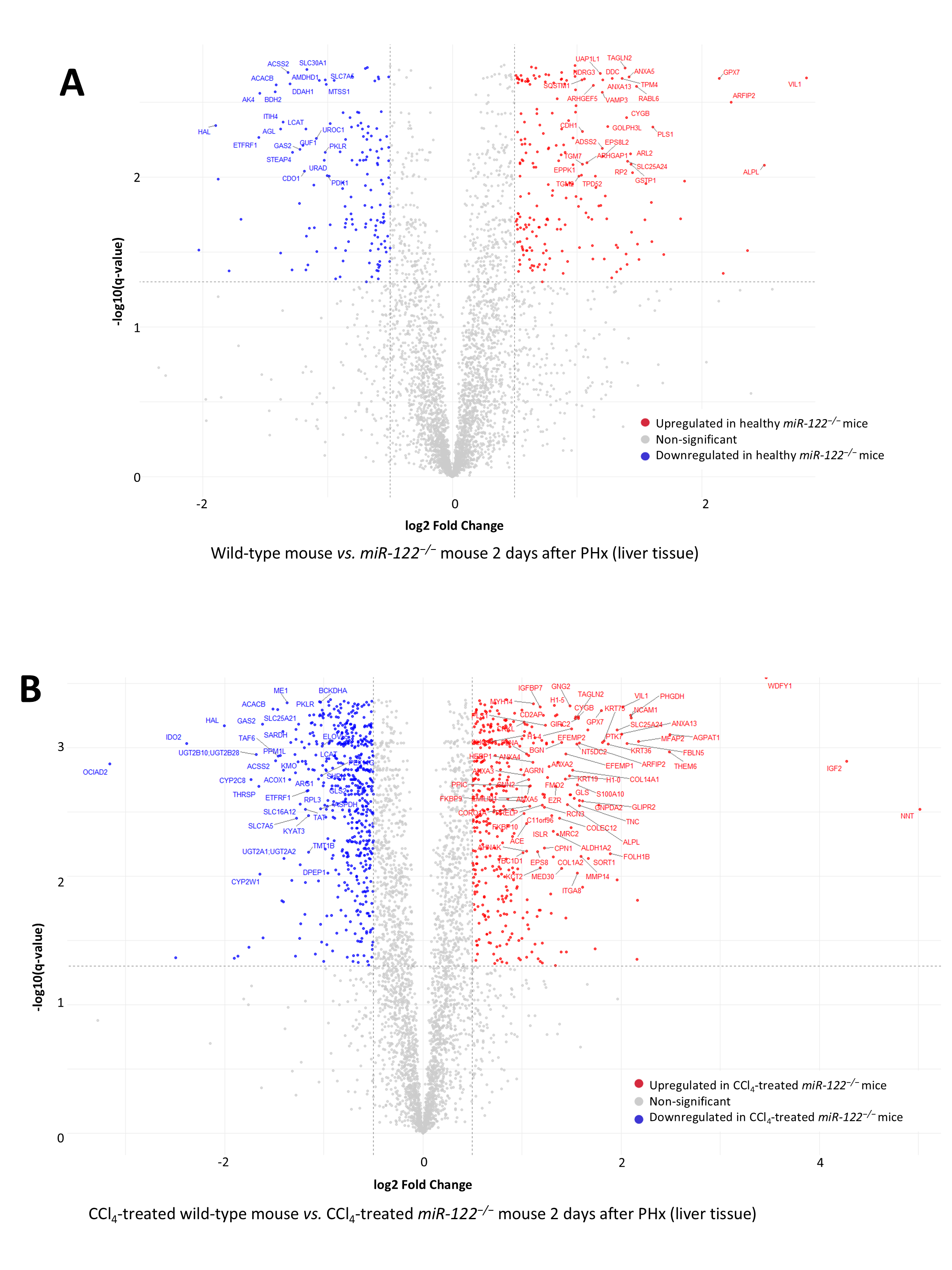

Supplement: Supplementary file 1 [file ijms-27-03149-s001.zip › Supplementari Figure S4 png.PNG]

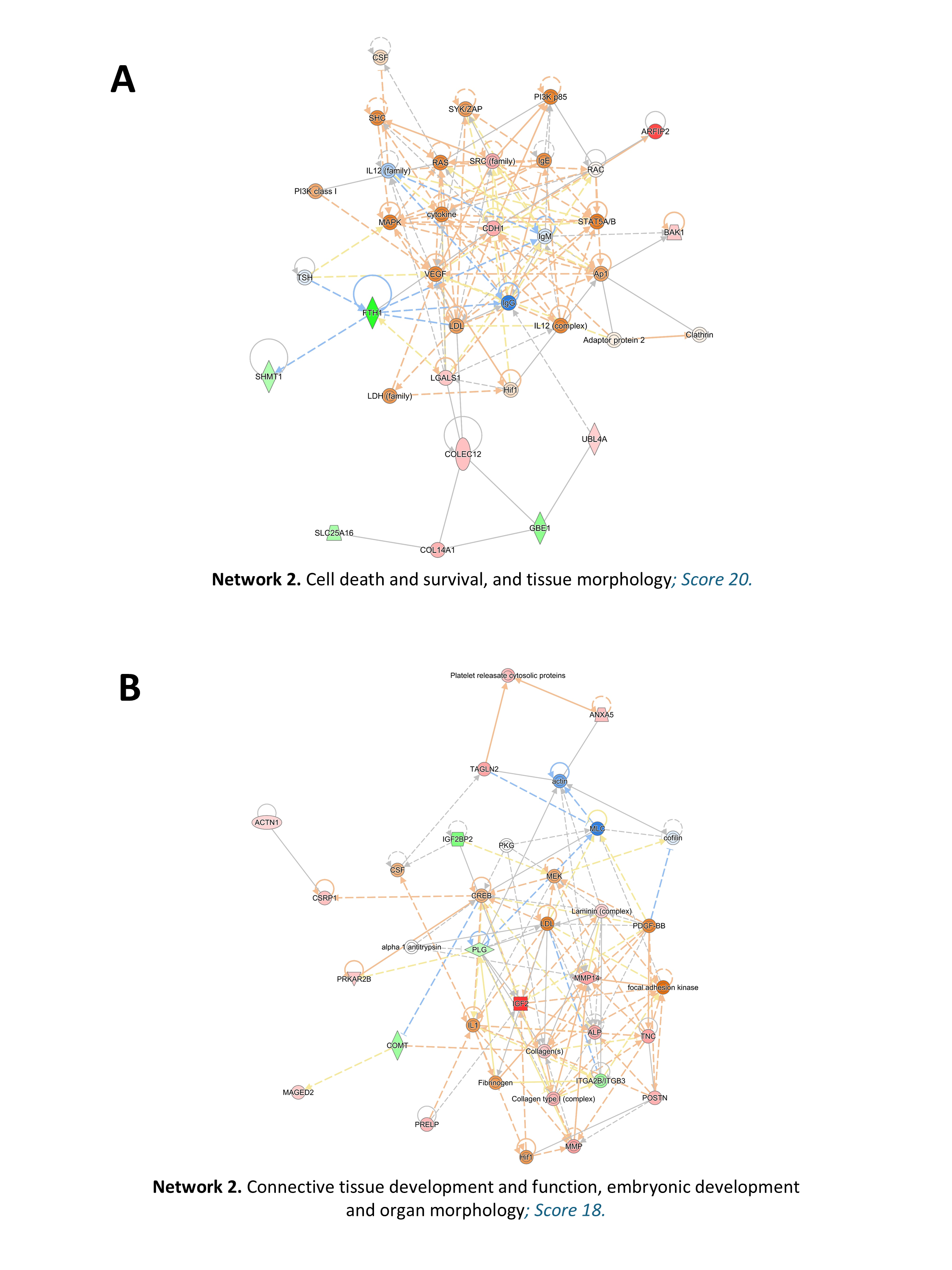

Supplement: Supplementary file 1 [file ijms-27-03149-s001.zip › Supplementari Figure S5 png.PNG]

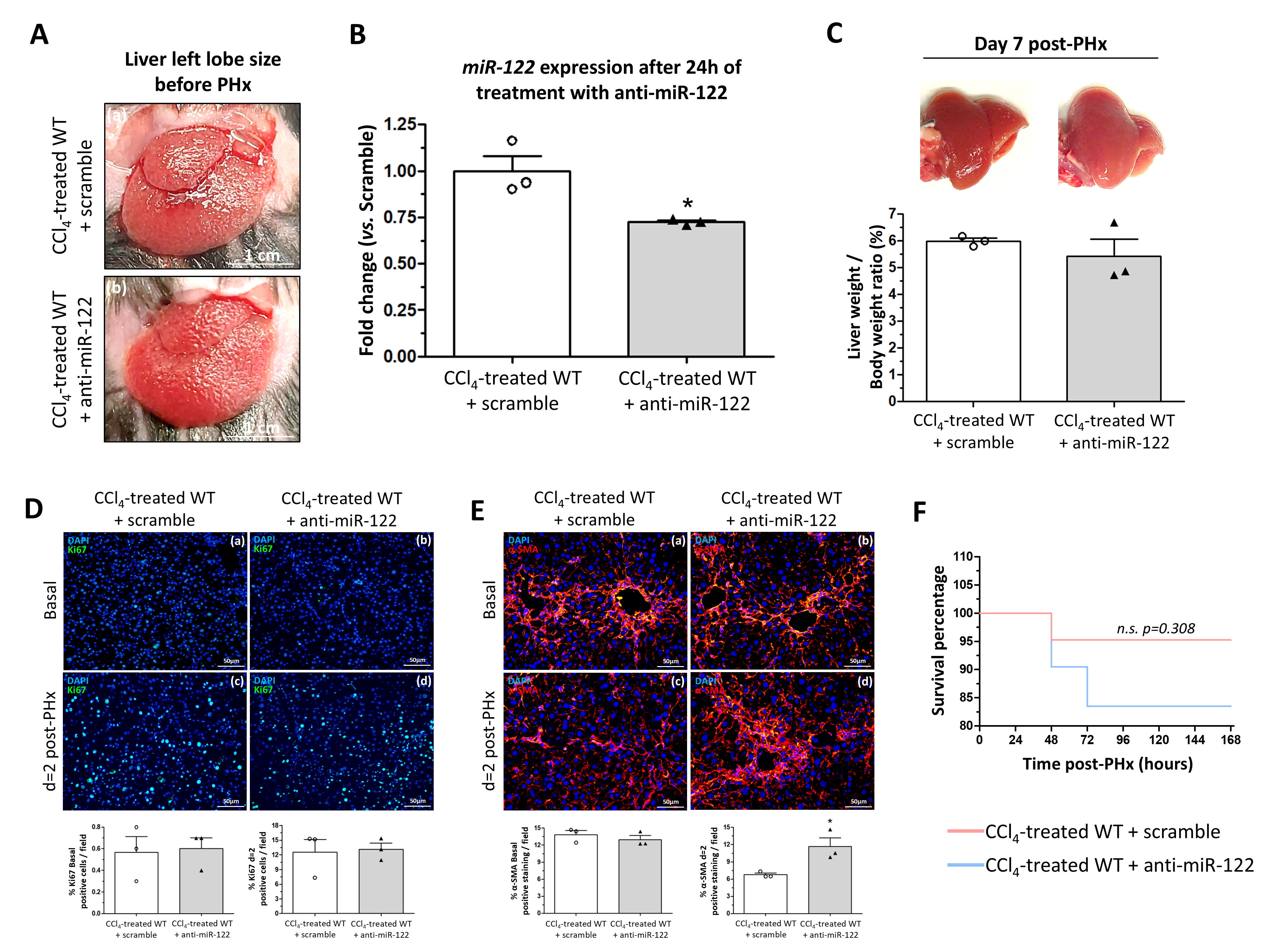

Supplement: Supplementary file 1 [file ijms-27-03149-s001.zip › Supplementary Figure S3 png.PNG]

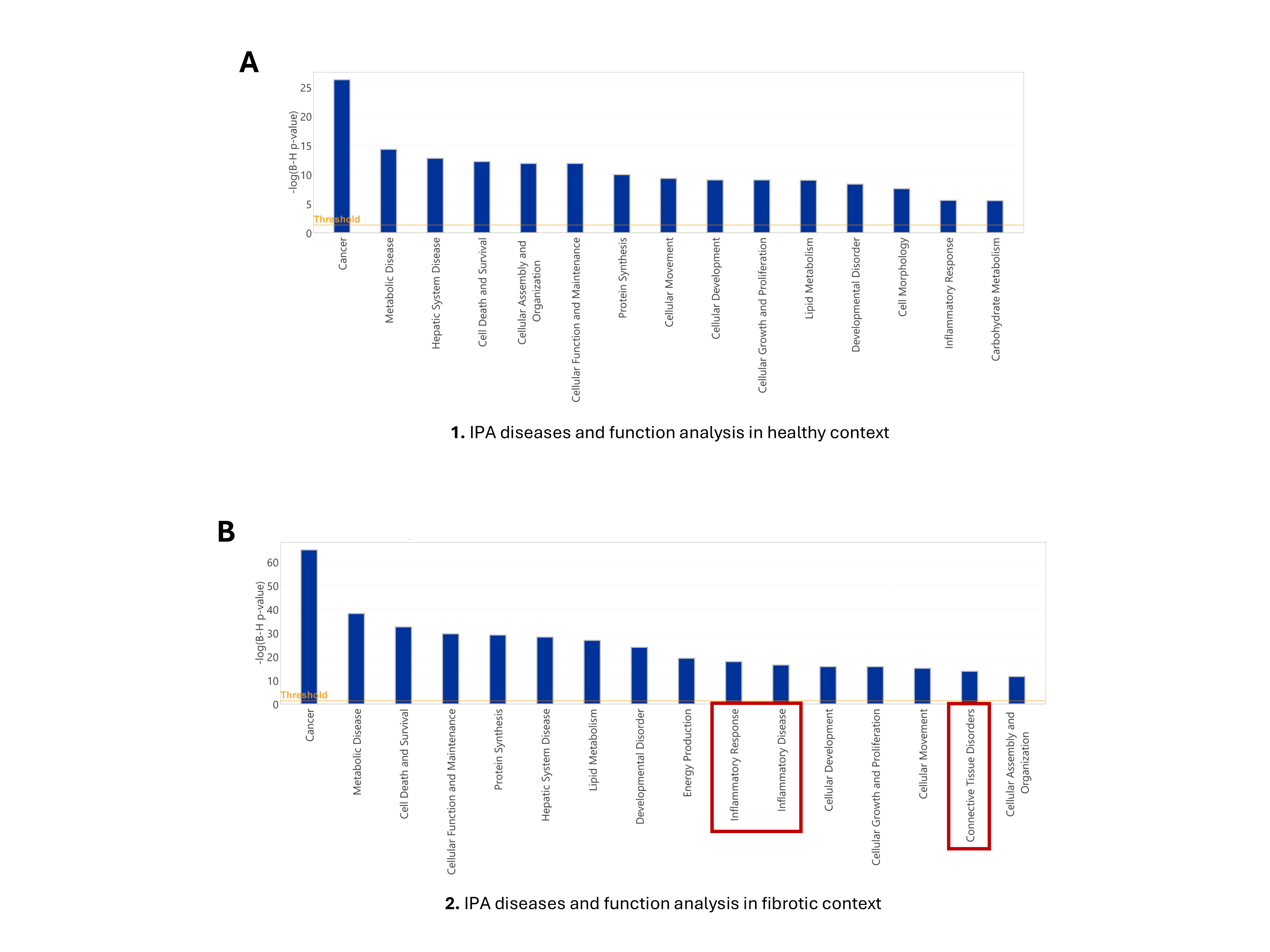

Supplement: Supplementary file 1 [file ijms-27-03149-s001.zip › Supplementary Figure S6 png.PNG]

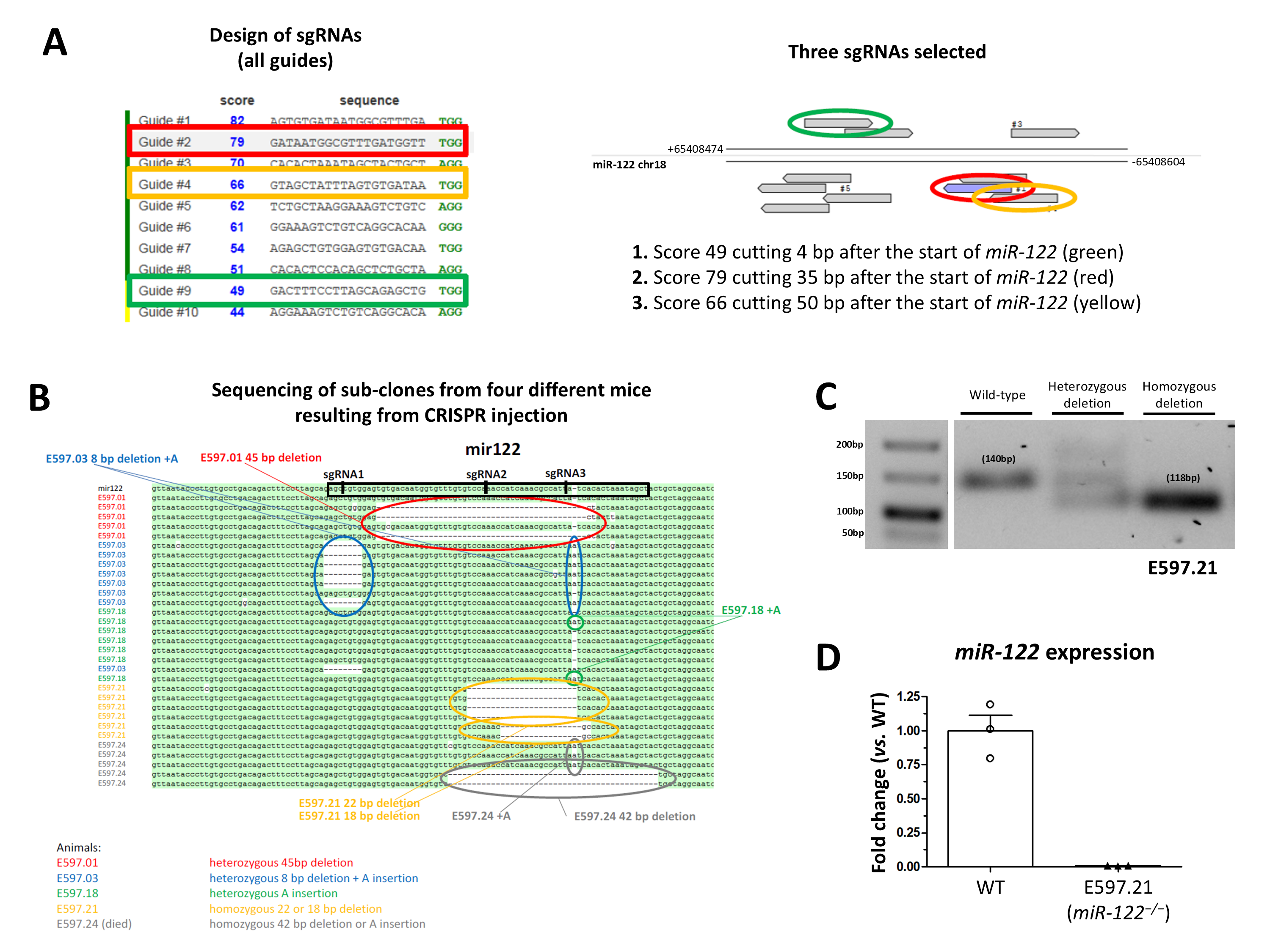

Supplement: Supplementary file 1 [file ijms-27-03149-s001.zip › Supplementary Figure S7 png.PNG]
